# Mesenchymal Stem Cells Polarize Macrophages to an Anti‐Inflammatory Phenotype to Ameliorate Diabetic Nephropathy

**DOI:** 10.1155/sci/6684410

**Published:** 2026-02-18

**Authors:** Linxi Zhang, Songyan Yu, Yu Cheng, Xiafang Lin, Zhengyuan Gong, Jing Xue, Bing Li, Yaqi Yin, Junyan Zou, Rui Wei, Tianpei Hong, Yiming Mu

**Affiliations:** ^1^ Department of Endocrinology and Metabolism, Peking University Third Hospital, Beijing, 100191, China, puh3.net.cn; ^2^ Department of Endocrinology, Beijing Tiantan Hospital, Capital Medical University, Beijing, 100070, China, ccmu.edu.cn; ^3^ Department of Endocrinology, The First Medical Center of Chinese PLA General Hospital, Beijing, 100853, China, 301hospital.com.cn; ^4^ Department of Endocrinology, Xinqiao Hospital, The Second Affiliated Hospital of Army Medical University, Chongqing, 400037, China, xqhospital.com.cn

**Keywords:** diabetic nephropathy, fibrosis, inflammation, macrophages, mesenchymal stem cell

## Abstract

In diabetic nephropathy (DN), classically activated macrophages (M1) are significantly increased, whereas alternatively activated macrophages (M2) are markedly decreased in the renal tissues. Mesenchymal stem cells (MSCs) have been shown to stimulate macrophages from M1 phenotype to M2 phenotype. Thus, we aimed to investigate whether the polarization of M1/M2 induced by MSCs was involved in DN. We injected human umbilical cord MSCs (UC‐MSCs) into DN rats and found UC‐MSC infusion reduced the infiltration of M1 macrophages and increased the infiltration of M2 macrophages in the glomerulus, thereby attenuating histopathological renal damage and improving renal inflammation and fibrosis in DN rats. Then, peritoneal macrophages were extracted and directed into M1 macrophages by lipopolysaccharides (LPS) in vitro. After coculturing UC‐MSCs with M1 macrophages, we found that the M1 macrophage markers and related pro‐inflammatory cytokines decreased. However, the expression of the M2 macrophage markers, as well as the anti‐inflammatory cytokines, increased observably. Furthermore, UC‐MSCs increased the expression of interleukin‐4 receptor alpha chain (IL‐4Rα) on macrophages by secreting interleukin‐6 (IL‐6); blocking IL‐6 secretion inhibited the effect of UC‐MSCs on M2 macrophage polarization. Then, we explored the mechanism by which M2 macrophages ameliorate DN in vitro and found that UC‐MSC‐induced M2 macrophages attenuated the secretion of the chemokine monocyte chemoattractant protein‐1 (MCP‐1) in hyperglycemia‐induced mesangial cells, which led to reduced macrophage recruitment and infiltration. Moreover, UC‐MSC‐induced M2 macrophages inhibited transforming growth factor β (TGF‐β) in glomerular mesangial cells. Our study proposes and discusses a mechanism by which MSCs promote the polarization of macrophages from M1 into M2 in the kidney, thereby ameliorating DN.

## 1. Introduction

Diabetic nephropathy (DN) is one of the most serious complications of diabetes mellitus, occurs in 20%–40% of diabetic patients, and is the most common cause of end‐stage chronic kidney disease [1–3]. DN is morphologically characterized by thickening of the glomerular basement membrane (GBM), mesangial expansion, and glomerulosclerosis, leading to proteinuria, hypertension, and decreased glomerular filtration rate. Despite current treatment may prevent or delay the development of DN, the identification of new methods for the treatment of DN based on the pathophysiological mechanism is necessary.

Evidence has suggested that macrophages, as key inflammatory cells, played a crucial role in the pathogenesis of DN [4–6]. In human progressive DN, macrophages accumulate within glomeruli and interstitium, and the intensity of the macrophage infiltration is associated with the rate of subsequent decline in renal function [6]. Another study demonstrated that monocyte chemoattractant protein‐1 (MCP‐1, also known as CCL2)‐mediated macrophage accumulation and activation played a critical role in the development of DN in streptozotocin (STZ)‐induced mouse [5]. Macrophages are characterized as M1 (classically activated macrophages) and M2 (alternatively activated macrophages) phenotypes. Classically activated M1 macrophages are broadly characterized as being pro‐inflammatory, while alternatively activated M2 macrophages are involved in tissue repair and remodeling [7–10]. M1 macrophages are positively correlated with the progression of DN; in contrast, M2 macrophages exert a protective effect on renal function in DN [11–15]. Therefore, promoting the polarization of renal macrophages toward the M2 phenotype may represent a novel therapeutic strategy for DN.

Mesenchymal stem cells (MSCs) have previously been reported to halt the progression of DN by improving the inflammatory microenvironment, but the underlying mechanism remained elusive [16]. Notably, MSCs have diverse potential therapeutic applications for different organs and tissues via interactions with components of both the innate and adaptive immune systems. MSCs have been demonstrated to promote the polarization of macrophages from the primarily pro‐inflammatory M1 phenotype to the anti‐inflammatory M2 phenotype both in vitro and in vivo [17–19]. Our previous study demonstrated that MSCs promoted M2 polarization to alleviate insulin resistance and repair β‐cell function [20–23]. However, the role of MSCs in modulating macrophage polarization in DN has not been reported. Therefore, in the current study, we explored the mechanism by which human umbilical cord MSCs (UC‐MSCs) promoted the polarization of macrophages from M1 to the M2 phenotype in the kidney, thereby ameliorating DN. Our findings provide a theoretical basis for the therapeutic potential of MSCs on DN.

## 2. Materials and Methods

### 2.1. Animals

Eight‐week‐old male SD rats were fed a high‐fat diet (HFD) or a normal‐chow diet (NCD) for 8–9 weeks. Randomization was used to allocate experimental units to the control and treatment groups. After 8–9 weeks of feeding, when the body weight of SD rats in the high‐fat feeding group reached about 600 g, STZ was injected intraperitoneally at a dose of 22 mg/kg. After STZ injection, the random blood glucose levels of the tail tip of the rats were measured every other day, and the random blood glucose ≥16.7 mmol/L for three consecutive days was defined as diabetes. Intraperitoneal glucose tolerance tests (IPGTTs) and insulin tolerance tests (IPITTs) were performed to confirm type 2 diabetes mellitus (T2DM) (Supporting Information 1: Figure S1). This study strictly adheres to the 3R principles of animal ethics. To achieve the research objectives while avoiding excessive animal sacrifice, the sample size was selected based on previous literatures [20, 21]. In our study, 24 rats were used in the experiment, which were planned to be divided into three groups: the MSC group, DN group, and N group. Among them, eight rats in the control group were fed an NCD, while 16 rats were fed an HFD to establish diabetic models. Of the 16 rats, 14 successfully established the diabetic model and were subsequently randomly assigned to two groups: the MSC group and the DN group. Additionally, seven rats were randomly selected from the eight rats in the control group. Block randomization was adopted to balance the age and weight of rats in each group. Rats in the DN group were subsequently fed an HFD for another 8 weeks to mimic the early stages of human DN. A total of 3 × 10^6^ MSCs suspended in 0.5 mL of phosphate‐buffered saline (PBS) were infused via the tail vein every 2 weeks (referred to as the MSC group), whereas DN group and NCD (N group) rats were infused with PBS as a control. The treatments were performed four times in total. The selection of therapeutic doses of UC‐MSCs and infusion protocols was based on the experimental results of previous studies [20, 21, 24–26]. At 15 weeks after STZ injection, IPGTTs and IPITTs were performed again to assess the effects of UC‐MSCs. A hyperinsulinemic–euglycemic clamp study was performed to observe the effects of UC‐MSCs in modulating insulin sensitivity. For hyperinsulinemic–euglycemic clamp study, 4 mU/kg/min insulin was intravenously administered into fasted rats at a rate of 2 mL/min to obtain euglycemia. Blood glucose levels were monitored at 10 min intervals. Adjust glucose‐infusion rate (GIR) as required until a steady state is achieved. All in vivo procedures were approved by the Ethics Committee of the First Medical Center of Chinese PLA General Hospital. To minimize investigator bias, researchers carrying out the studies were blinded. Different researchers were responsible for specified sets of experiments. Researchers tasked with the preparation of MSCs and PBS were required to code these reagents, allocate them to experimental animals or cell cultures, and were excluded from the subsequent administration of the coded reagents to the animals or cells. Researchers responsible for injecting MSCs or PBS into the animals were unaware of the animals’ experimental groups and performed the injections solely based on the codes of the injected reagents and the animals’ ear tags. Pathological sections and image results were labeled with numbers rather than according to the groups. Researchers tasked with histological scoring and data analysis conducted the analysis in a blinded manner, with no prior knowledge of the experimental groups, and they were not involved in animal or cell experiments.

### 2.2. Cell Culture

Human umbilical cords were obtained from healthy women who gave birth at the Chinese PLA General Hospital. A table with demographics on umbilical cord donors is present in Supporting Information 2: Table S1. All subjects provided informed consent. Ethics committee of the First Medical Center of Chinese PLA General Hospital approved the study. After informed consent, fresh umbilical cords were collected and processed as soon as possible from healthy women who delivered in the Chinese PLA General Hospital. Under sterile conditions, the cords were rinsed three times by PBS. The cords were cut into 2–3 cm pieces and squeezed with clean sterile to remove the blood. The cords were washed twice. Peel off the umbilical cords (avoid taking too thick a layer), then remove the arteries and vein to isolate Wharton’s jelly from the umbilical cord. The umbilical cords were cut into 1 mm^3^ pieces with sterile eye shears and evenly spread on a 15 cm dish. Let them stand for 15 min, 15 mL of serum‐free medium rewarmed in advance was added. The dishes were placed in a cell incubator at 37°C in 5% CO_2_. The medium was replaced 5 days after the initial plating. After 2–3 days, the cells were subcultured at a ratio of 1:1 and diluted 10 times with 0.25% trypsin to digest the cells. After 3 days, when the cells grew to 70%–80% confluence density, the cells were passaged at a ratio of 1:4. Cells can be used only after passage 4–5.

Peritoneal macrophages were obtained from SD rats by peritoneal lavage with H‐DMEM (Gibco, USA) for 7–10 min. The purity of macrophages determined via anti‐F4/80 immunofluorescence staining exceeded 90%. After 1 × 10^5^ peritoneal macrophages were seeded onto six‐well plates for 12 h, 1 μg/mL lipopolysaccharides (LPS; Sigma–Aldrich) was added for 24 h. The macrophages were then cultured with 3 × 10^4^ UC‐MSCs in a trans‐well system for 48 h. For interleukin‐6 (IL‐6) neutralization experiments, 0.1 μg/mL IL‐6 neutralizing antibody (NA; R&D Systems, USA) was added to the culture medium when UC‐MSCs were cocultured with macrophages. IgG control antibody was used for control. The UC‐MSCs and culture medium IL‐6 levels were detected by quantitative real‐time reverse transcriptase polymerase chain reaction (qRT‐PCR) and enzyme‐linked immunosorbent assay (ELISA) to confirm the successful blockade of IL‐6.

Rat glomerular mesangial cells (HBZY‐1) were purchased from the Basic Medical Cell Center (Institute of Basic Medicine, Chinese Academy of Medical Sciences). HBZY‐1 cells were cultured in L‐DMEM (Gibco, USA) supplemented with 10% FBS for 12 h, and then the medium was replaced with H‐DMEM (Gibco, USA) containing 10% FBS. After 12 or 24 h of stimulation, increasing interleukin‐1β (IL‐1β), transforming growth factor β (TGF‐β), and collagen I/IV levels were measured to mimic the glucotoxicity of glomerular mesangial cells.

The human monocytic cell line Tohoku Hospital Pediatrics−1 (THP‐1) cells were purchased from the American Type Culture Collection (ATCC, Manassas, VA, USA). THP‐1 cells were cultured in RPMI‐1640 containing 10% FBS at 37°C, in a 5% CO_2_ atmosphere. The macrophage‐like M0 cells were induced by treatment with phorbol 12‐myristate 13‐acetate (PMA, 160 ng/mL, Sigma) for 24 h. The nonadherent cells were removed with PBS. Adherent cells were further incubated with fresh medium containing LPS (100 ng/mL, Sigma) and interferon γ (IFN‐γ, 50 ng/mL, Prime Gene BioTech, Shanghai, China) to stimulate M1 macrophage polarization. Subsequently, the conditioned medium was removed. The macrophages were then cultured with 3 × 10^4^ UC‐MSCs in a trans‐well system for 48 h to stimulate M2 macrophage polarization.

Human renal mesangial cells (HRMCs) were purchased from the ATCC (Manassas, VA, USA) and maintained in DMEM including 10% FBS at 37°C, in a 5% CO_2_ atmosphere. HRMCs were incubated with high glucose (30 mM) medium. After 12, 24, 48, and 72 h of increased glucose, we changed the normal glucose (5.6 mM) medium and detected the expression of TGF‐β and collagen I. Then after 48 h’ high glucose stimulation, HRMCs were cocultured with UC‐MSC‐induced M2 macrophages.

### 2.3. Biochemical Tests for Albuminuria

Urine from each animal was collected via a metabolic cage system. The rats were housed in metabolic cage for 24 h, during which they were free to eat and drink water under constant temperature and humidity. There were two funnels for separation of urine and feces. The urine collection funnel was well sealed to prevent urine evaporation. Albuminuria levels were measured using immune‐turbidimetric methods (Chondrex 9040), and creatinine levels were measured using enzymatic methods (Yaji Biological YS03735B). The urinary albumin levels were normalized to the urinary creatinine levels (albumin creatine ratio [ACR]).

### 2.4. Immunohistochemistry Staining and Immunofluorescence Staining

The right kidney obtained from experimental animal after anesthesia was removed for immunoblotting and qRT‐PCR tests, and the animal was perfused with 4% paraformaldehyde through the aortic trunk cannulated by the left ventricle. The fixed kidney was embedded in paraffin, and then 10‐μm‐thick sections were cut and stained with hematoxylin and eosin (H&E), Masson, periodic acid Schiff (PAS), and Sirius red according to standard protocols. The Sirius red staining images have two types of images. One was observed under the optical microscope, and another black background was observed under a polarizing light microscope. Under the polarized light, the ordered complexes formed after Sirius red staining cause birefringence of light. Different collagen types produce unique interference colors due to differences in fiber diameter, usually appearing red (Collagen I), green (Collagen III), or pale blue (Collagen IV). Glomerular damage was expressed as the percentage of glomeruli presenting mesangial expansion and glomerulosclerosis. The glomerulosclerosis index showed the mesangial matrix expansion or sclerosis levels, and it was performed as described previously [27, 28]. Briefly, each glomerulus on a single section was graded from 0 to 4+, where 0 represents no lesion, and 1, 2, 3, and 4+ represent mesangial matrix expansion or sclerosis, involving ≤25, 25–50, 50–75, or >75% of the glomerular tuft area, respectively. For staining of collagen I/IV, fibronectin, IL‐1β, TNF‐α, TGF‐β, CD68, CD206, CD11c, and CD163, kidney samples were immersed in 4% paraformaldehyde, and paraffin‐embedded sections were incubated with primary antibodies and biotinylated secondary antibody (Supporting Information 2: Table S2). The positively stained area of the images was calculated by Image Pro Plus 6.0 software (Microsoft Media Cybernetics, USA). The area of positively stained region represented the mean density of each image in different groups of renal tissue.

### 2.5. Kidney Ultrastructure Evaluation

Electron microscopy was used to evaluate the ultrastructure of GBM and the podocytic processes. Fresh kidney tissue was fixed in 1% glutaraldehyde, followed by 1% osmium tetroxide and uranyl acetate, and finally embedded in epoxy resin. The specimens were examined and photographed using a transmission electron microscope (JEM‐1400EX, Japan) at 3000, 5000, and 30000 × magnification at an accelerating voltage of 80 kV. Electron micrographs were randomly taken from three glomeruli per kidney, and the kidneys were also randomly taken from three rats of each group.

### 2.6. Immunoblotting

The proteins extracted from tissues and cells were assessed by Western blotting; 10% SDS‐polyacrylamide electrophoresis and nitrocellulose membranes were used. The membranes were blocked with 5% nonfat milk and incubated with primary antibodies (Supporting Information 2: Table S3) at 4°C overnight, followed by incubation with a secondary antibody. β‐Actin was used as a loading control for comparison between samples. Image J software (NIH, USA) was used to analyze the blots.

### 2.7. Quantitative Real‐Time Reverse Transcriptase Polymerase Chain Reaction

Total RNA from tissues and cultured cells was extracted using TRIzol reagent (Life Technologies, 15596018, USA) and a reverse transcription kit (Thermo Fisher, K1622, USA) in accordance with the manufacturer’s protocols. An ABI Prism thermal cycler (model StepOne‐Plus; Applied Biosystems, CA, USA) and SYBR Green PCR Master Mix (Applied Biosystems) were used to quantify target genes (Supporting Information 2: Table S4). β‐Actin was used as the internal control. Data represent three independent experiments.

### 2.8. ELISA

The protein levels of TNF‐α‐stimulated gene 6 (TSG‐6), indoleamine 2,3‐dioxygenase (IDO), IL‐6, and TGF‐β secreted by UC‐MSCs were quantified via commercial ELISA kits (NeoBioscience, China) according to the manufacturer’s instructions.

### 2.9. Statistical Analysis

Data are presented as mean ± SD from at least three independent experiments. Statistical analyses were performed using one‐way ANOVA followed by Tukey’s HSD post hoc test. Two‐tailed *p*  < 0.05 was considered statistically significant (SPSS 19.0, IBM Corp., USA).

## 3. Results

### 3.1. UC‐MSC Therapy Improved Glucose Homeostasis and Inhibited the Increase of Albuminuria in Diabetic Rats

Osteogenic and adipogenic differentiation demonstrated a multilineage capacity comparable with UC‐MSCs (Figure 1A). The flow cytometry analysis showed that UC‐MSCs expressed high levels of surface CD73, CD90, and CD105 but lacked surface CD45, CD34, and HLA‐DR (Figure 1B). The T2DM rat model was induced with a combination of an 8–9 week HFD and a single intraperitoneal injection of a low dose of STZ (Supporting Information 1: Figure S1). The HFD was fed to diabetic rats for a further 8 weeks to mimic the early stage of human DN. A total of 3 × 10^6^ UC‐MSCs suspended in 0.5 mL of PBS were infused via the tail vein every 2 weeks (referred to as the MSC group), whereas rats in the DN group and NCD (N group) were infused with PBS as a control. The treatments were performed four times in total (Figure 1C). After 4 weeks of treatment, the random blood glucose level in the MSC group was 387.45 ± 20.70 mg/dL, which was lower than the 446.40 ± 30.93 mg/dL in the DN group (Figure 1D). Compared with the DN group, body weight in the MSC group increased more markedly (Figure 1E). IPGTTs and IPITTs (Figure 1F,G) showed significant deterioration of glucose disposal and insulin sensitivity in T2DM rats, which were markedly alleviated in the rats receiving UC‐MSC administration. Additionally, the results of the hyperinsulinemic–euglycemic clamp study showed a significantly increased GIR in the MSC‐treated group (Figure 1H).

Figure 1UC‐MSCs therapy improved glucose homeostasis and inhibited the increase in albuminuria in diabetic rats: (A) capacity for differentiation to osteoblasts and adipocytes. Scale bar, 100 μm; (B) the fluorescence‐activated cell sorting (FACS) analysis of UC‐MSC surface molecules showed high levels of CD73, CD90, and CD105, and the absence of CD45, CD34, and HLA‐DR; (C) experimental protocol for UC‐MSC therapy in high‐fat diet diabetic rats; (D) random blood glucose levels were detected at 4, 8, 10, 12, 14, and 15 weeks after STZ infusion; (E) body weight levels were measured at 4, 8, 12, and 15 weeks after STZ infusion; (F) blood glucose concentration of three groups after IPGTTs; (G) insulin tolerance was evaluated by IPITTs; (H) glucose‐infusion rate during hyperinsulinemic–euglycemic clamp analysis of three groups; and (I) the ACR was assessed before and after UC‐MSCs treatment. Results were presented as the means ± SD. *n* = 7 rats per group.  ^∗^ *p* < 0.05,  ^∗∗^ *p* < 0.01.

**Figure:**
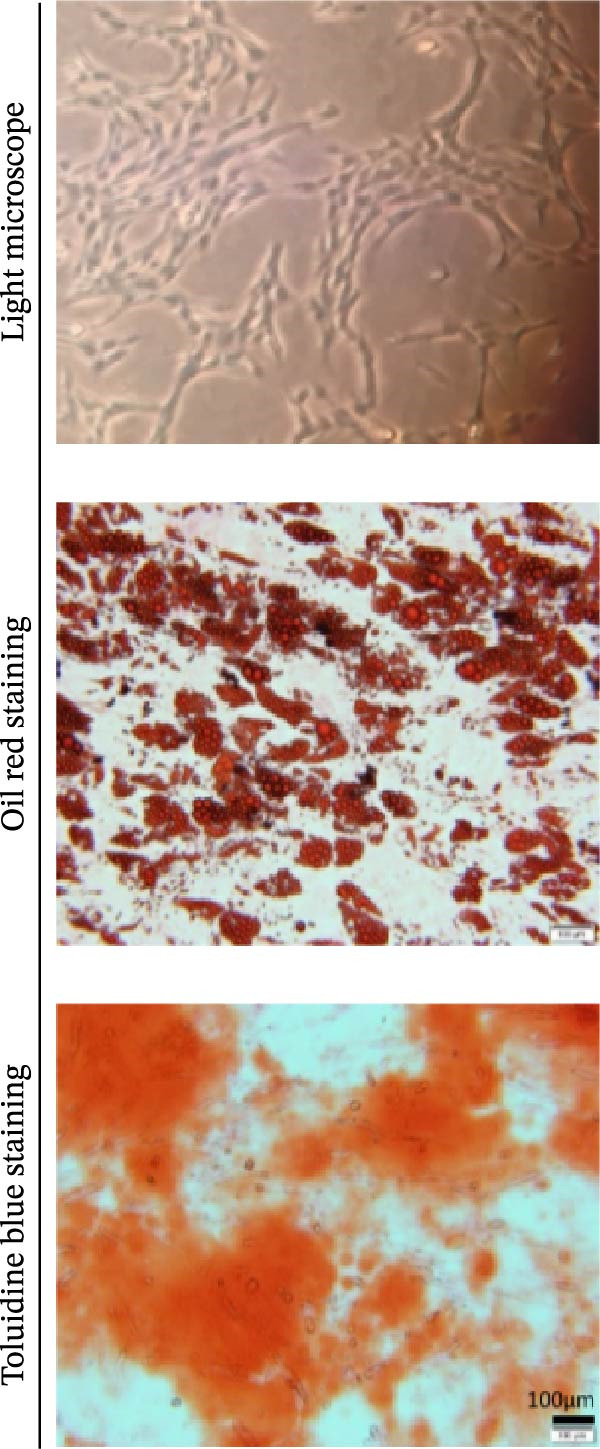
(A)

**Figure:**
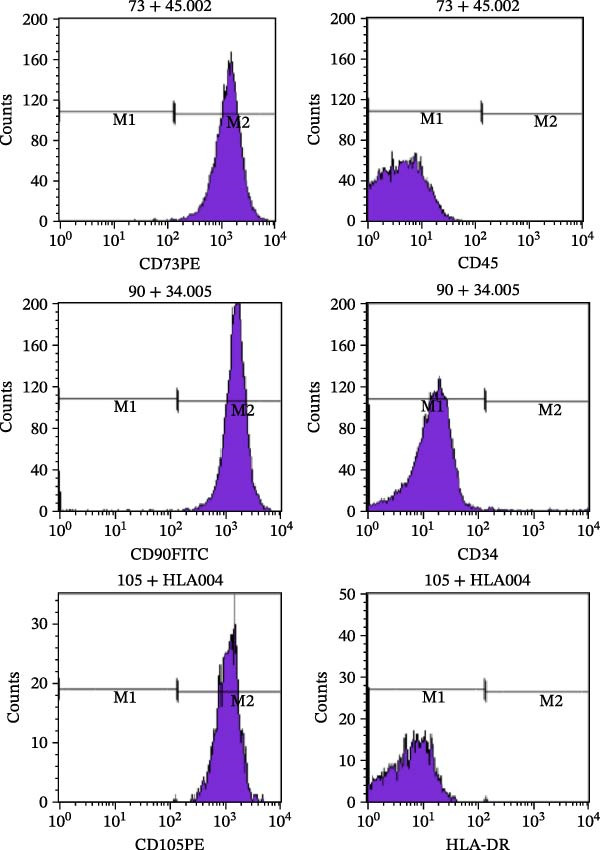
(B)

**Figure:**
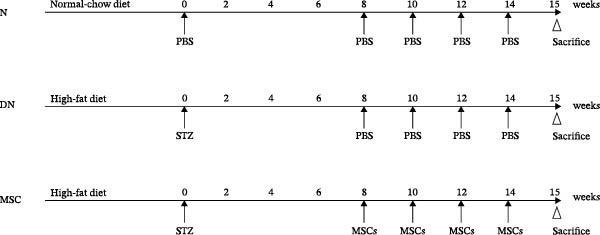
(C)

**Figure:**
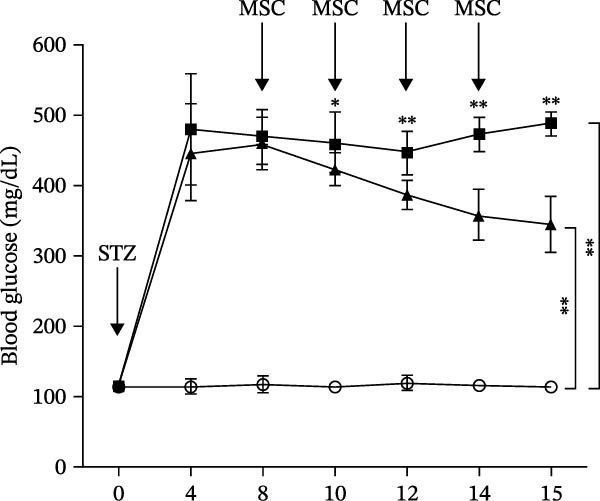
(D)

**Figure:**
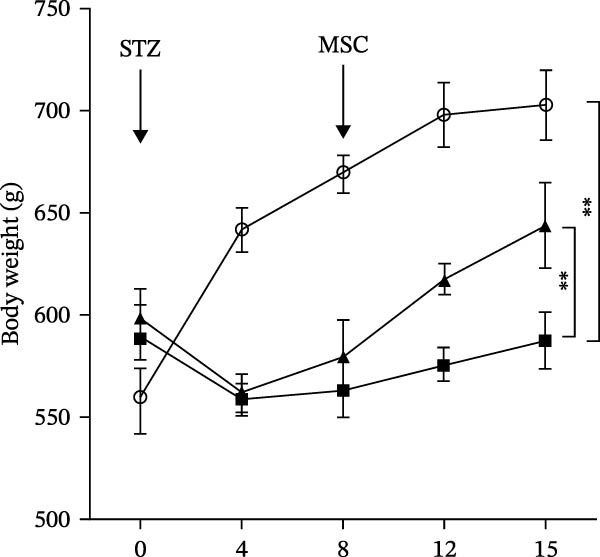
(E)

**Figure:**
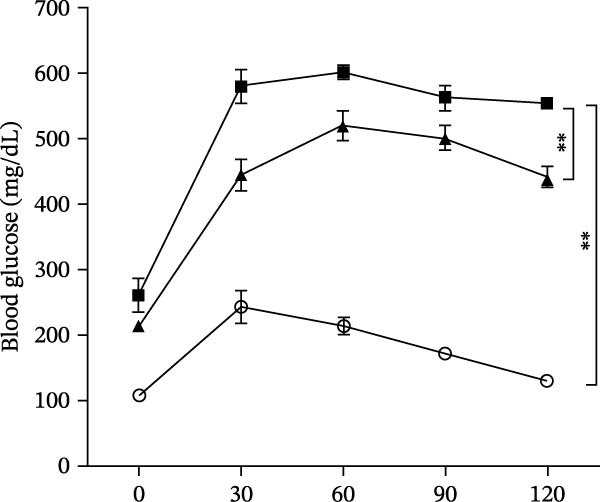
(F)

**Figure:**
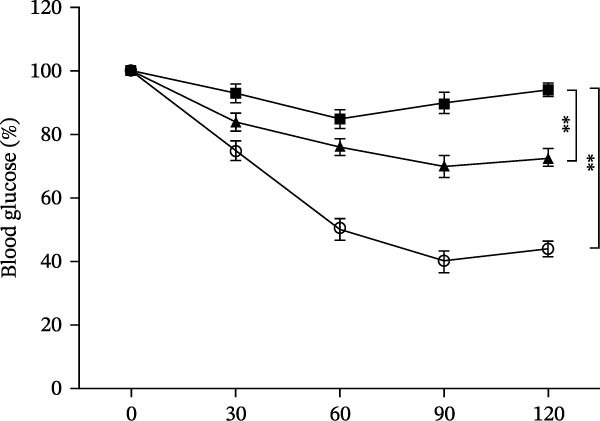
(G)

**Figure:**
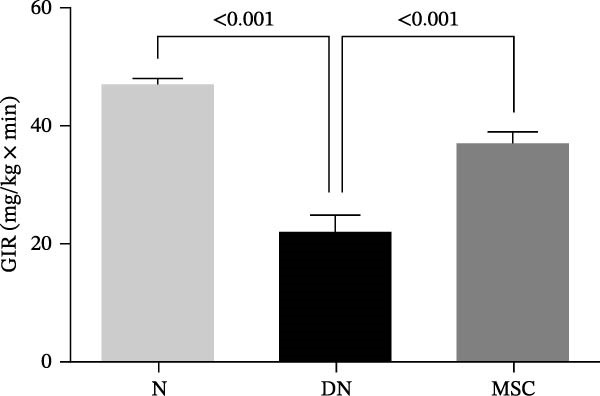
(H)

**Figure:**
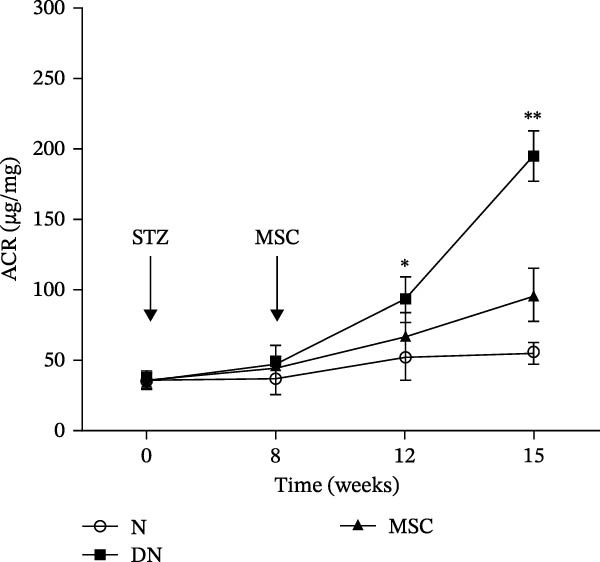
(I)

Urine was collected via a metabolic cage system after STZ injection and MSC treatment at a fixed time. The ACR after STZ injection and before MSC treatment showed no significant difference in each group. The ACR in the DN group was elevated slightly at 4 weeks after MSC administration (*p* = 0.032) and elevated significantly at 7 weeks after MSC administration (*p* = 0.001) compared with that in the MSC treatment group (Figure 1I), revealing MSC infusions effectively inhibited the progressive increase in albuminuria in diabetic rats.

### 3.2. UC‐MSC Therapy Attenuated Histopathological Damage in Diabetic Rats

H&E staining demonstrated that DN group rats had notable glomerular hypertrophy, glomerulosclerosis, and mesangial matrix expansion compared with the normal group and MSC group rats. Masson staining showed renal fibrosis, and PAS staining showed fibrous protein, mesangial matrix, and amyloid protein in diabetic rats; nevertheless, these changes were markedly reduced in the MSC treatment group. Sirius red staining further demonstrated that renal fibrosis was significantly reduced in the MSC group compared with the DN group (Figure 2A,B). Transmission electron microscopy (TEM) was used for the ultrastructural assessment of glomerular injury (Figure 2C). Consistent with our histologic findings, TEM indicated mesangial matrix deposition, local podocyte effacement, GBM thickening, and endothelial cell proliferation in diabetic rats, whereas UC‐MSC treatment ameliorated these pathological alterations dramatically.

Figure 2UC‐MSCs therapy attenuated histopathological damage in diabetic rats: (A,B) histological characteristics of the kidney sections in H&E, Masson, PAS, and Sirius red staining after MSC administration. Scale bar, 20 μm. The scores of histological staining were calculated from at least five sections of each rat. *n* = 7 rats per group. Results were presented as the means ± SD. (C) Transmission electron micrographs of kidney samples are shown (magnification: × 3000, × 5000, × 30,000.).

**Figure:**
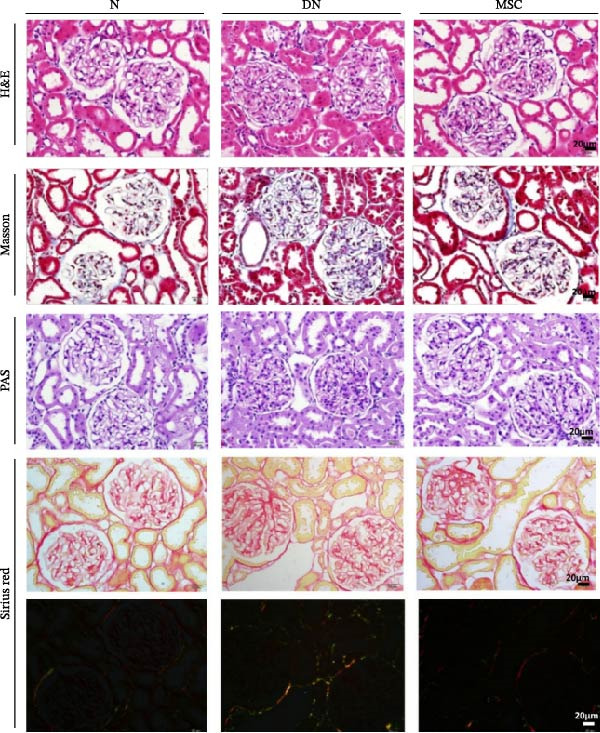
(A)

**Figure:**
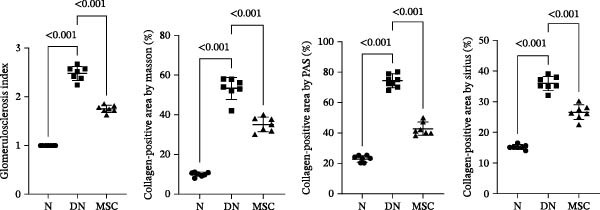
(B)

**Figure:**
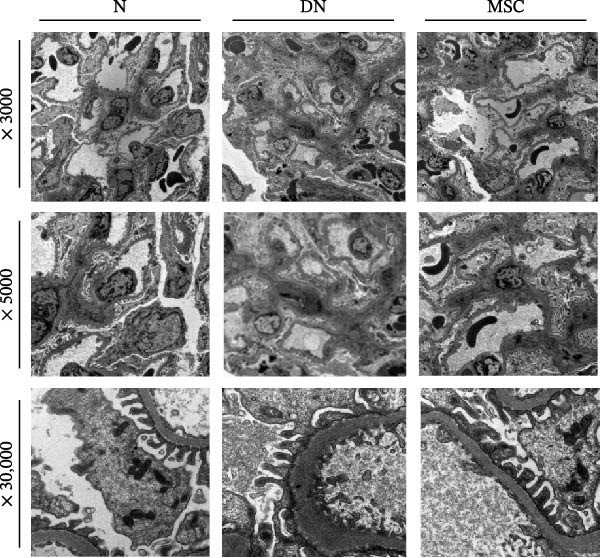
(C)

### 3.3. UC‐MSC Therapy Ameliorated Renal Fibrosis in Diabetic Rats

Renal fibrosis is the final outcome of progressive DN. To investigate the impact of UC‐MSCs on renal fibrosis, we detected the expression of the main extracellular matrix (ECM) components, including collagen I, collagen IV, and fibronectin, in kidney by immunohistochemical staining. The ECM component‐positive area in the UC‐MSC therapy rats was significantly reduced compared with that in diabetic rats (Figure 3A,B). The protein expression of collagen I decreased; moreover, the mRNA expression of collagen I, collagen IV, and α‐smooth muscle actin (α‐SMA) declined remarkably after UC‐MSC therapy in the MSC group compared with the DN group (Figure 3C,D). In summary, these results demonstrate that UC‐MSC infusion reduced renal fibrosis.

Figure 3UC‐MSCs therapy ameliorated renal fibrosis in diabetic rats: (A,B) the expression of collagen I, collagen IV, and fibronectin in the kidney was determined by immunohistochemistry assays. Scale bar, 20 μm. Quantification was calculated from at least five sections of each rat; (C) immunoblotting analysis of collagen I was tested. The ratios of collagen I to β‐actin were quantitated. Results are presented relative to those of the normal group, set as 1; (D) collagen I, collagen IV, and α‐SMA mRNA expression were evaluated by real‐time PCR. Results are presented relative to those of the normal group, set as 1; results were presented as the means ± SD. *n* = 7 rats per group.

**Figure:**
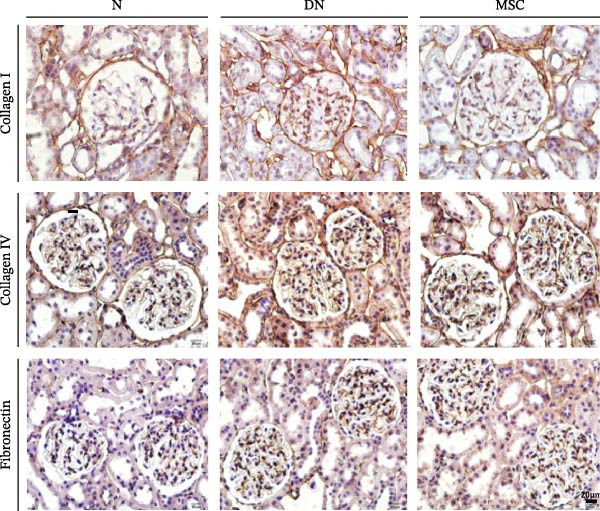
(A)

**Figure:**
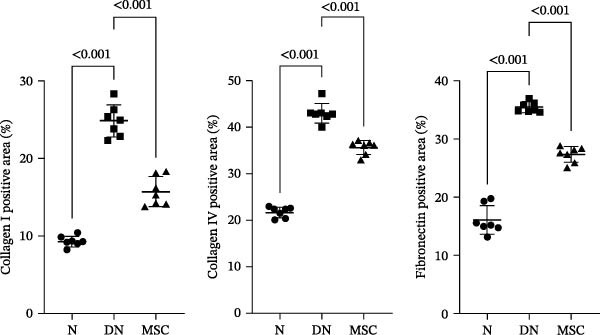
(B)

**Figure:**
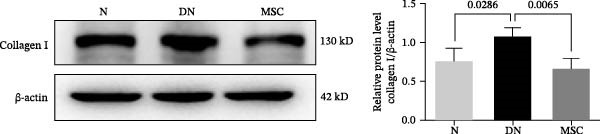
(C)

**Figure:**
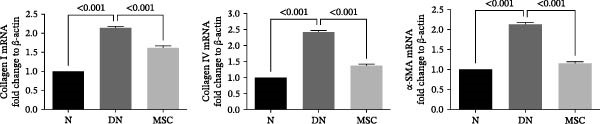
(D)

### 3.4. UC‐MSC Therapy Ameliorated Renal Inflammation in Diabetic Rats

Considerable evidence indicated that inflammation played a critical role in renal fibrosis, accelerating the progression of DN. We measured pro‐inflammation cytokines, which were well‐known markers of DN. Immunohistochemistry staining revealed that the positive staining areas of IL‐1β, TNF‐α, and TGF‐β were significantly reduced in the MSC group compared with the DN group (Figure 4A,B). Additionally, Western blotting showed reduced protein expression of TNF‐α and TGF‐β after UC‐MSC administration (Figure 4C). The mRNA expression of pro‐inflammation cytokines, including TNF‐α, TGF‐β, IL‐1β, prostaglandin E receptor 4 (EP‐4), and signal transducer and activator of transcription 3 (STAT3), was downregulated by UC‐MSC therapy compared with the no‐therapy condition. In addition, UC‐MSC promoted anti‐inflammatory cytokine IL‐10 mRNA upregulation in the kidney (Figure 4D), suggesting that UC‐MSCs have strong immunosuppressive effects on DN.

Figure 4UC‐MSCs therapy ameliorated renal inflammation in diabetic rats: (A,B) immunohistochemistry analysis of the expression of IL‐1β, TNF‐α, and TGF‐β in the kidney. Scale bar, 20 μm. Quantification was calculated from at least five sections of each rat; IL‐1β, TNF‐α, and TGF‐β are expressed in both glomeruli and renal tubules, and our analytical focus is on glomerular expression. (C) Immunoblotting analysis of TGF‐β and TNF‐α in the kidney. Relative protein levels are quantified by the ratio of TGF‐β to β‐actin and TNF‐α to β‐actin; (D) TNF‐α, TGF‐β, IL‐10, IL‐1β, EP‐4, and STAT3 mRNA expressions were evaluated by real‐time PCR. Results are presented relative to those of the normal group, set as 1; results were presented as the means ± SD. *n* = 7 rats per group.

**Figure:**
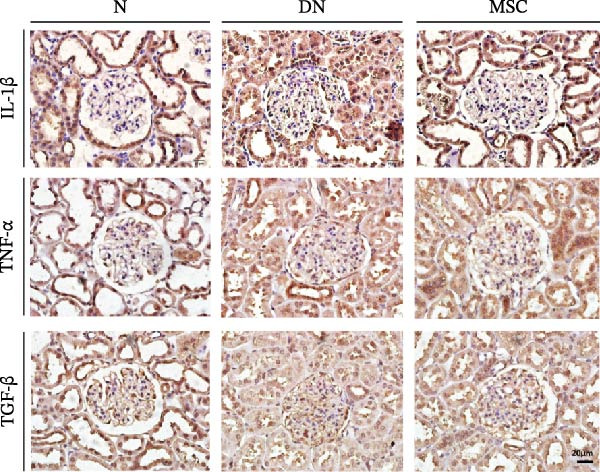
(A)

**Figure:**
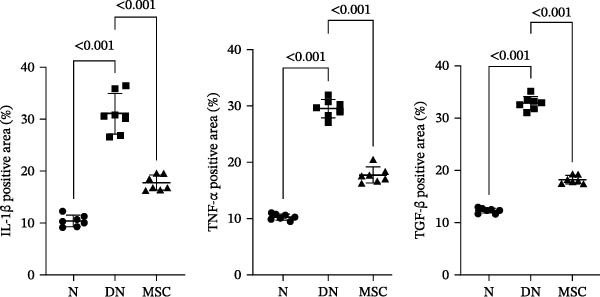
(B)

**Figure:**
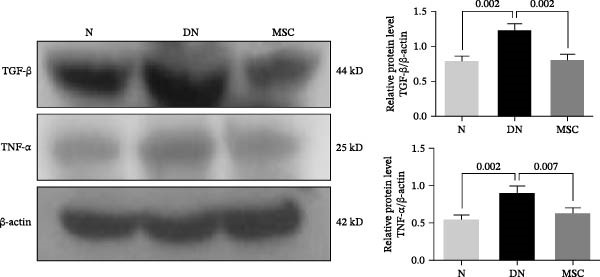
(C)

**Figure:**
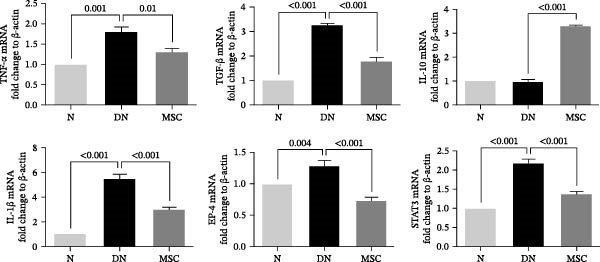
(D)

### 3.5. UC‐MSC Therapy Induced M2 Macrophage Polarization in the Kidney of Diabetic Rats

Macrophages, as key inflammatory cells accumulating within glomeruli, are associated with renal damage in DN. Therefore, we investigated the effects of UC‐MSCs on macrophage polarization in diabetic rats. Immunohistochemical staining revealed that there were more CD68‐positive macrophages (regardless of sub‐phenotype) and more CD11c (an M1 marker)‐positive macrophages in the DN group than in the MSC group. However, there were more CD206 (an M2 marker)‐positive macrophages and more CD163 (an M2 marker)‐positive macrophages in the MSC group than in the DN and N groups (Figure 5A,B). The mRNA expression of MCP‐1, which is involved in macrophage infiltration, was elevated in diabetic rats. In addition, RT‐PCR analysis showed that the mRNA expressions of CD68, inducible nitric oxide synthase (iNOS, an M1 marker), and CD206 were consistent with the results of immunohistochemical staining, demonstrating that UC‐MSCs reduced the infiltration of total macrophages and induced M2 macrophage phenotype polarization in the kidney of diabetic rats (Figure 5C).

Figure 5UC‐MSCs therapy induced M2 macrophage polarization in the kidney in diabetic rats: (A,B) immunohistochemical staining of CD68 (marker for total macrophages), CD206 and CD163 (markers for M2 macrophages), and CD11c (marker for M1 macrophages) in the kidney of rats from the N, DN, and MSC groups. The arrow points to positive cells. Scale bar, 20 μm. Five nonconsecutive sections from each rat with seven rats in each group, resulting in a total of 35 sections per group for quantitative analysis. All sections were imaged under standardized microscopic conditions, and positive cells were counted using ImageJ software with consistent threshold settings. (C) CD68, MCP‐1, iNOS, and CD206 mRNA expressions were evaluated by real‐time PCR. Results are presented relative to those of the normal group, set as 1; results were presented as the means ± SD. *n* = 7 rats per group.

**Figure:**
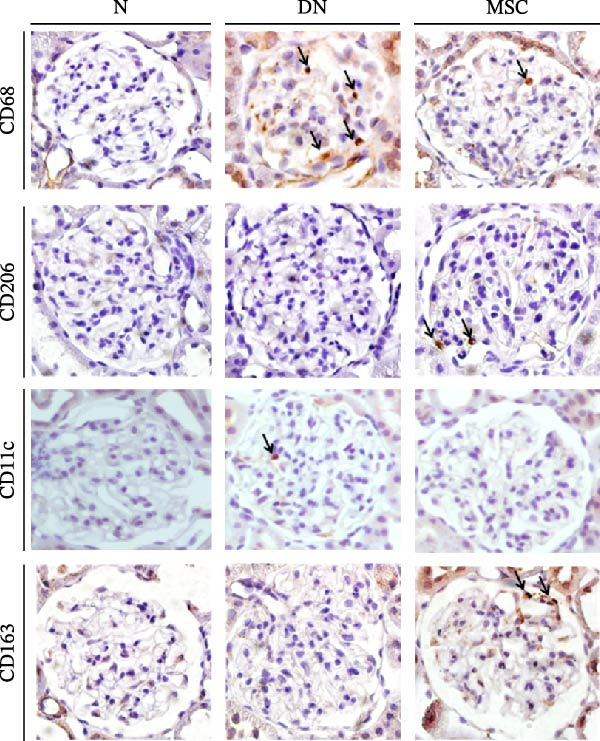
(A)

**Figure:**
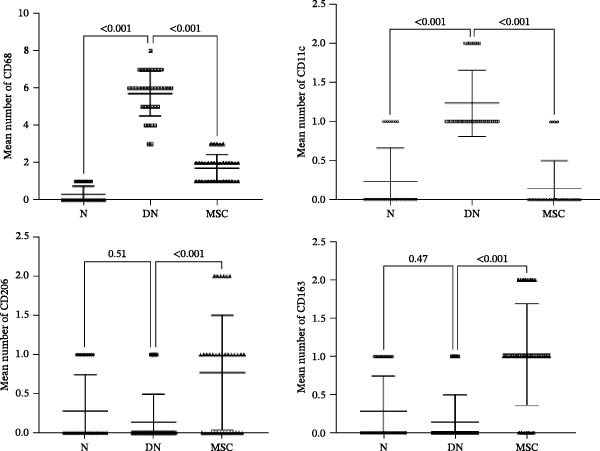
(B)

**Figure:**
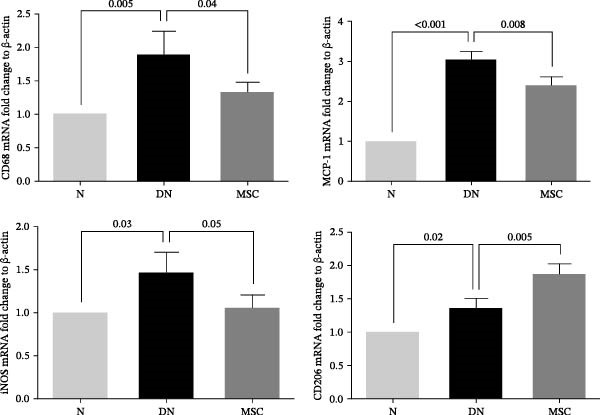
(C)

### 3.6. UC‐MSCs Suppressed M1 Macrophage Polarization and Induced M2 Macrophage Polarization In Vitro

To confirm the effects of UC‐MSCs on M2 macrophage polarization, we extracted peritoneal macrophages from SD rats and verified that more than 95% of cells were F4/80‐positive (a marker for macrophages regardless of sub‐phenotypes) (Figure 6A). Light microscopy revealed that LPS administration induced the extension of many pseudopodia by macrophages. After coculture with UC‐MSCs, fewer macrophage pseudopodia were observed in vitro (Figure 6B). LPS administration induced peritoneal macrophages to express more iNOS (a marker for M1) and less Arginase 1 (Arg 1, a marker for M2) than coculture with UC‐MSCs, suggesting that LPS polarized peritoneal macrophages to M1 phenotypes, whereas UC‐MSCs suppressed M1 polarization and induced M2 macrophages polarization. The Western blotting results also showed a higher expression level of Arg 1 in the UC‐MSCs coculture group than in the LPS group (Figure 6C,D). Consistently, the mRNA expression of the M1 macrophage maker NOS2 and the related pro‐inflammatory cytokines TNF‐α, TGF‐β, and IL‐1β decreased after UC‐MSCs coculture. The mRNA expression of the M2 macrophage markers CD163 and CD206 and anti‐inflammatory cytokine IL‐10 increased observably in the MSC group (Figure 6E).

Figure 6UC‐MSCs suppressed M1 macrophage polarization and induced M2 macrophage polarization in vitro: (A) peritoneal macrophages were extracted, and it was verified that more than 95% of the cells were F4/80 positive (red, a marker for macrophages regardless of sub‐phenotypes). Scale bar, 50 μm; (B) under a light microscope, LPS administration was observed to induce the extension of many pseudopodia by macrophages. After coculture with UC‐MSCs, fewer macrophage pseudopodia were observed in vitro. Scale bar, 50 μm; (C) immunofluorescence of Arg 1 and iNOS in the control, LPS, and MSC groups. Scale bar, 100 μm. Quantification of Arg 1 positive or iNOS positive macrophages was determined by evaluating at least five random fields of each section; representative of three independent experiments; data are presented as mean ± SD from three independent experiments; individual replicate values are provided in Supporting Information 2: Table S5. (D) Immunoblotting analysis of Arg 1 in the three groups. Relative protein level is quantified by ratio of Arg1 to β‐actin; representative of three independent experiments; data are presented as mean ± SD from three independent experiments; quantitative data are presented in Supporting Information 2: Table S5. (E) iNOS, CD163, CD206, IL‐10, TNF‐α, TGF‐β, and IL‐1β mRNA expression were evaluated by real‐time PCR. Results are presented relative to those of the control group, set as 1; results were presented as the means ± SD.

**Figure:**
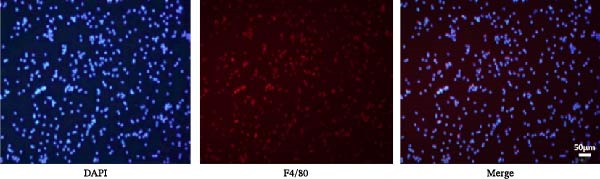
(A)

**Figure:**
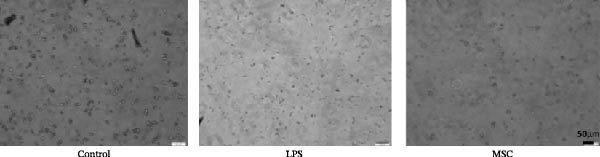
(B)

**Figure:**
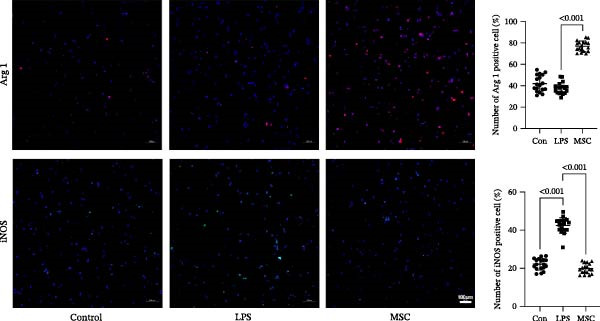
(C)

**Figure:**
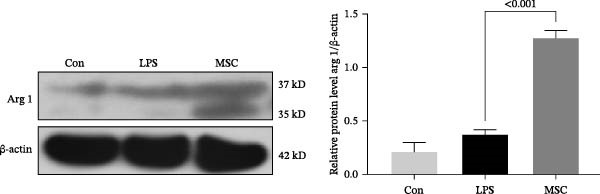
(D)

**Figure:**
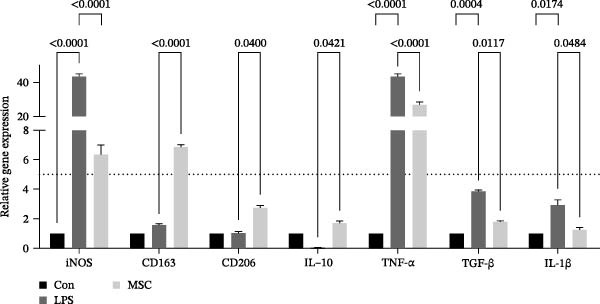
(E)

### 3.7. UC‐MSCs Induced M2 Macrophage Polarization via IL‐6/Interleukin‐4 Receptor (IL‐4R) In Vitro

Previous studies have shown that MSC‐mediated polarization of M2 macrophages depends on the secretion of prostaglandin E2 (PGE2), TSG‐6, IL‐6, IDO, and TGF‐β1 [21, 23, 29]. We analyzed the mRNA expression of these genes in UC‐MSCs to investigate the possible factors responsible for M2 macrophage polarization and found that only IL‐6 was significantly elevated (Figure 7A). When UC‐MSCs were cocultured with LPS‐stimulated macrophages from 12 to 48 h, the level of IL‐6 secreted from UC‐MSCs increased gradually (Figure 7B). Then, we used an IL‐6 NA to reduce IL‐6 to an extremely low level to determine whether IL‐6 induced M2 macrophage polarization (Figure 7C,D). According to the immunofluorescence results, Arg1 expression was markedly decreased after IL‐6 neutralization (Figure 7E,F). IL‐4 and IL‐13 have been demonstrated to be the major cytokines mediating M2 macrophage polarization via IL‐4R alpha chain (IL‐4Rα) overexpression. Nevertheless, IL‐4/IL‐13 were extremely low (Supporting Information 3: Figure S2), and IL‐4Rα protein expression in macrophages was upregulated after MSC coculture but downregulated significantly after IL‐6 neutralization (Figure 7G). Together, these studies revealed that IL‐6/IL‐4Rα played an essential role in the effect of UC‐MSCs on M2 polarization.

Figure 7UC‐MSCs induced M2 macrophage polarization via IL‐6/IL‐4R in vitro: (A) UC‐MSCs were cultured with LPS‐stimulated macrophages (M1) for 24 h, and the gene expression of the factors secreted by UC‐MSCs was detected by quantitative RT‐PCR analysis. The control group was UC‐MSCs cultured alone; (B) quantitative RT‐PCR analysis of IL‐6 expression in UC‐MSCs after cocultured with LPS‐stimulated macrophages (M1) for 12, 24, 36, and 48 h. The control group was UC‐MSCs cultured alone; (C) UC‐MSCs were treated with IgG or IL‐6 neutralizing antibody or without treatment. Then, UC‐MSCs were cultured with LPS‐stimulated macrophages (M1). The control group was UC‐MSCs cultured alone. Quantitative RT‐PCR analysis of IL‐6 expression in each group. Results in A, B, and C are presented relative to those of the control group, set as 1; (D) enzyme‐linked immunosorbent assays of IL‐6 in the medium of UC‐MSCs cocultured with LPS‐stimulated macrophages (M1) for treatment with IgG or an IL‐6 neutralizing antibody. The control group was UC‐MSCs cultured alone; (E,F) immunofluorescence of Arg 1 positive macrophages from control, LPS‐stimulated macrophages, LPS‐stimulated macrophages cocultured with UC‐MSCs, LPS‐stimulated macrophages cocultured with UC‐MSCs which were treated with IgG, LPS‐stimulated macrophages cocultured with UC‐MSCs which were treated with IL‐6 neutralizing antibody. Scale bar, 50 μm. Quantification of Arg 1 positive macrophages was determined by evaluating at least five random fields of each section; representative of three independent experiments; data are presented as mean ± SD from three independent experiments; quantitative data are presented in Supporting Information 2: Table S5. (G) Immunoblotting analysis of IL‐4Rα in LPS‐stimulated macrophages, LPS‐stimulated macrophages cocultured with UC‐MSCs which were treated with IgG, LPS‐stimulated macrophages cocultured with UC‐MSCs which were treated with IL‐6 neutralizing antibody. Relative protein level is quantified by ratio of IL‐4Rα to β‐actin. Results are representative of three independent experiments; data are presented as mean ± SD from three independent experiments; quantitative data are presented in Supporting Information 2: Table S5.

**Figure:**
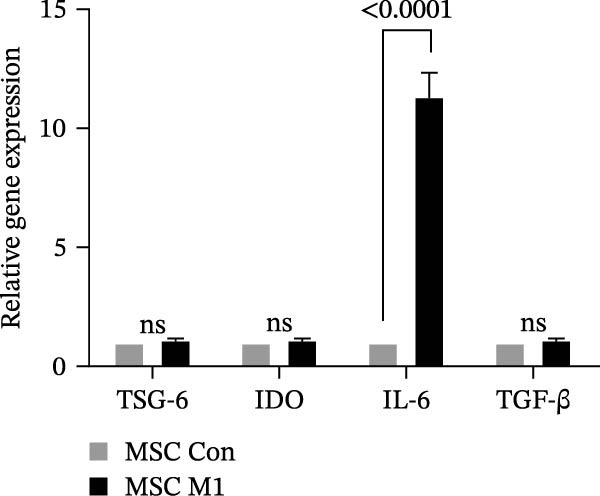
(A)

**Figure:**
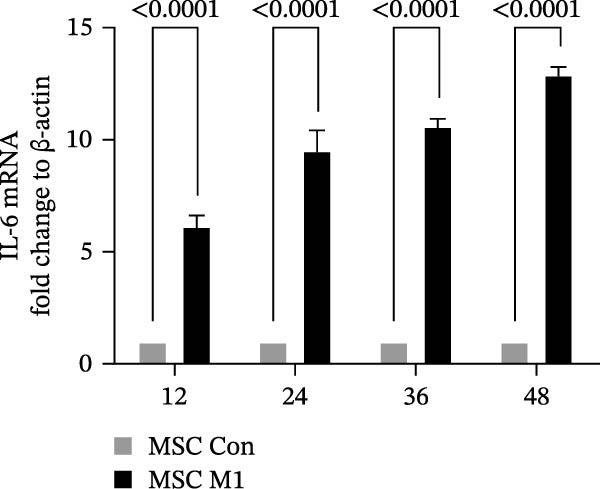
(B)

**Figure:**
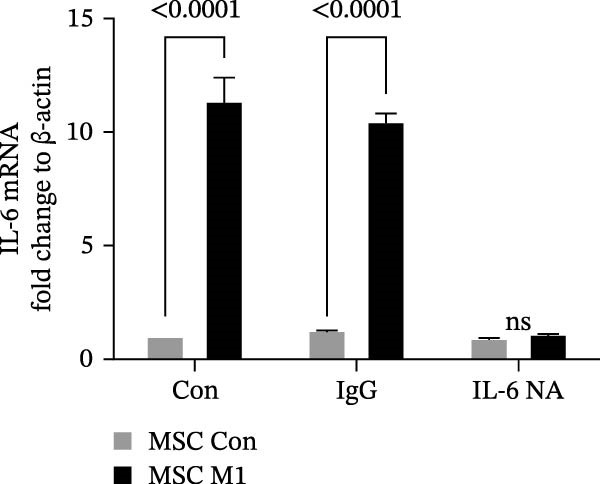
(C)

**Figure:**
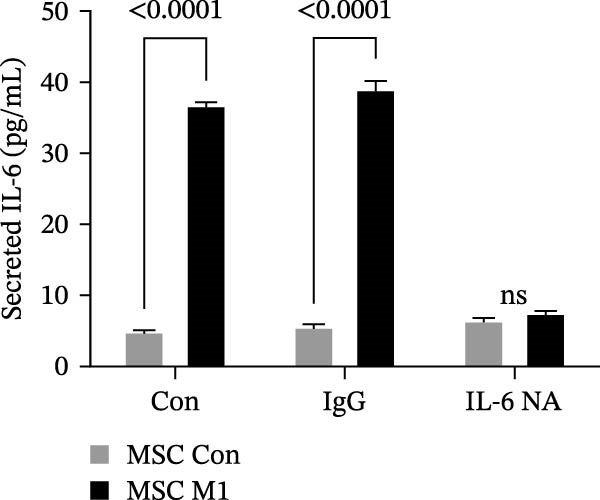
(D)

**Figure:**
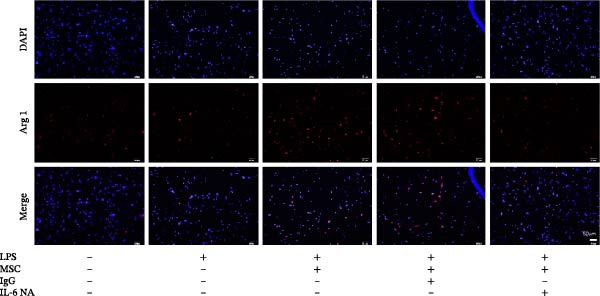
(E)

**Figure:**
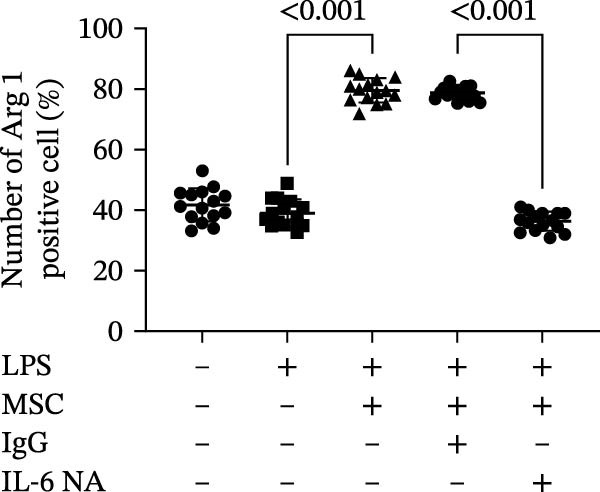
(F)

**Figure:**
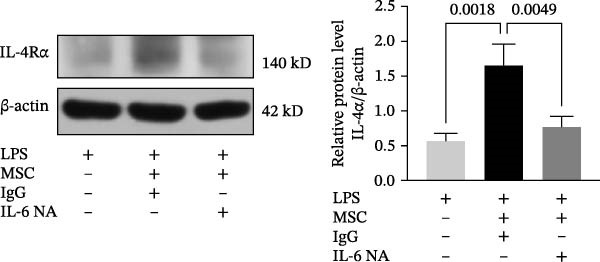
(G)

### 3.8. UC‐MSC‐Induced M2 Macrophages Protect HBZY‐1 From High‐Glucose Toxicity

To clarify the complex mechanisms by which UC‐MSC‐induced M2 macrophages improve the progression of DN, we used HBZY‐1 to further elucidate the mechanisms in vitro. According to the immunofluorescence analysis, compared with the other groups, the group cultured with high‐glucose DMEM for 24 h showed the highest collagen I expression (Figure 8A). Similarly, the mRNA expression of IL‐1β, TGF‐β, collagen I, and collagen IV was increased by culturing with high glucose concentrations for 24 h (Figure 8B). We next cocultured HBZY‐1 in high‐glucose DMEM with UC‐MSCs‐induced M2 macrophages in a trans‐well system. As expected, M2 macrophages markedly reduced collagen I expression compared with HBZY‐1 cultured in high‐glucose DMEM alone (Figure 8C). NADPH oxidase 4 (NOX‐4)/TGF‐β1 signaling activation mediated the accumulation of ECM [30]. UC‐MSC‐induced M2 macrophages downregulated NOX‐4, TGF‐β, and collagen I protein expression (Figure 8D) and reduced collagen I, collagen IV, IL‐1β, and CCL2 (involved in macrophage infiltration) mRNA expression (Figure 8E).

Figure 8UC‐MSCs‐induced M2 macrophages protect HBZY‐1 from high glucose toxicity: (A) HBZY‐1 cultured with L‐DMEM or H‐DMEM for 12 and 24 h was stained with anti‐collagen I (red) antibody. Scale bar, 75 μm. Quantification of collagen I positive was determined by evaluating at least five random fields of each section. Results are presented relative to those of the control group, set as 1; representative of three independent experiments; data are presented as mean ± SD from three independent experiments; quantitative data are presented in Supporting Information 2: Table S5. (B) Quantitative RT‐PCR analysis of IL‐1β, TGF‐β, collagen I, and collagen IV expression in HBZY‐1 cells. The results are presented relative to those of HBZY‐1 cultured with L‐DMEM for 12 h, set as 1; (C) photomicrographs of HBZY‐1 stained with anti‐collagen I (red) antibody from control (HBZY‐1 cultured with L‐DMEM for 24 h), HBZY‐1 cultured with H‐DMEM for 24 h, and HBZY‐1 cultured with H‐DMEM for 24 h cocultured with UC‐MSC‐induced M2 macrophages for 48 h groups. Scale bar, 25 μm. Quantification of collagen I positive macrophages was determined by evaluating at least five random fields of each section. Results are presented relative to those of the control group, set as 1; representative of three independent experiments; data are presented as mean ± SD from three independent experiments; quantitative data are presented in Supporting Information 2: Table S5. (D) Immunoblotting analysis of NOX‐4, TGF‐β, and collagen I in HBZY‐1 from the three groups. Protein levels are presented relative to β‐actin; representative of at least three independent experiments; data are presented as mean ± SD from three independent experiments; quantitative data are presented in Supporting Information 2: Table S5. (E) Quantitative RT‐PCR analysis of collagen I, collagen IV, CCL2, and IL‐1β gene expression in HBZY‐1 from the three groups. Results are presented relative to those of the control group, set as 1. Results were presented as the means ± SD.

**Figure:**
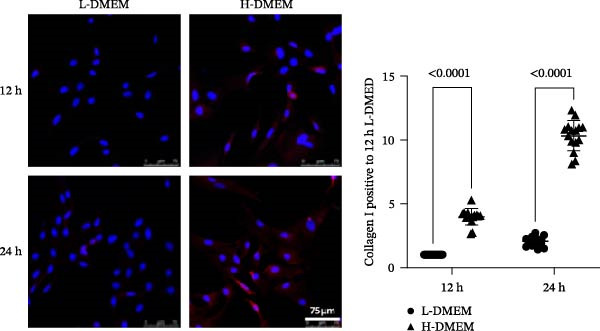
(A)

**Figure:**
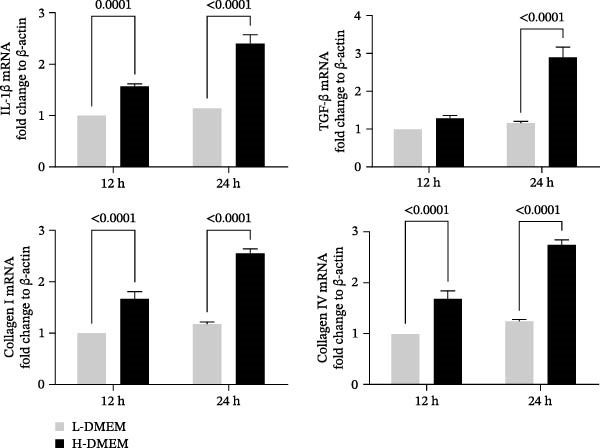
(B)

**Figure:**
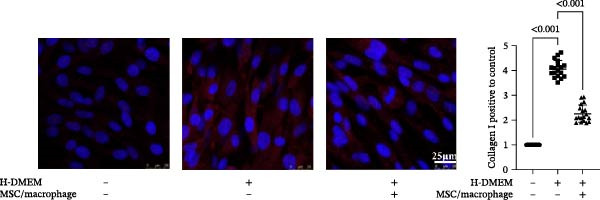
(C)

**Figure:**
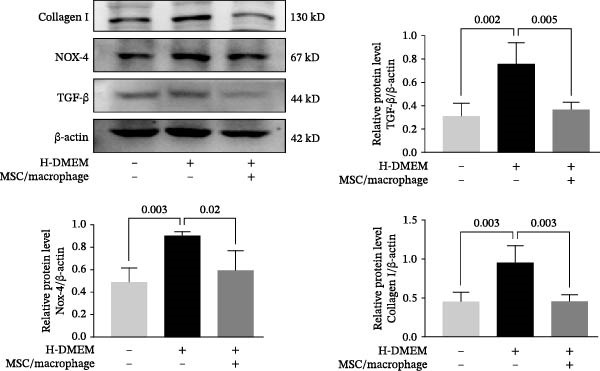
(D)

**Figure:**
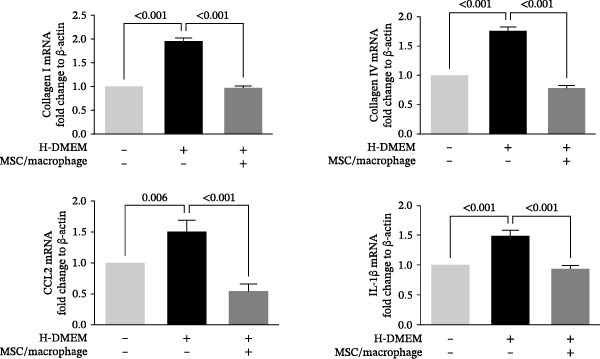
(E)

### 3.9. UC‐MSC‐Induced M2 Macrophages Ameliorated the ECM Accumulation in HRMC

The human monocytic cell line THP‐1 was induced by treatment with LPS and IFN‐γ to stimulate M1 macrophage polarization. Subsequently, the macrophages were then cultured with UC‐MSCs in a trans‐well system to stimulate M2 macrophage polarization. The cells were stained with CD80‐PE and CD206‐PE and then were analyzed by flow cytometry. LPS and IFN‐γ stimulation induced more CD80 (a marker for M1) expression, while UC‐MSCs induced more CD206 (a marker for M2) expression (Figure 9A). Quantitative RT‐PCR analysis of gene expression in THP‐1 macrophages from three groups revealed that LPS and IFN‐γ administration induced macrophages expressing more CD86 (a marker for M1) and the related pro‐inflammatory cytokines IL‐1β. After culturing with UC‐MSCs, macrophages expressed more CD206 (a marker for M2) and anti‐inflammatory cytokine IL‐10 and less CD86 (a marker for M1) and the related pro‐inflammatory cytokines IL‐1β. These results demonstrated that LPS and IFN‐γ polarized peritoneal macrophages to M1 phenotypes, whereas UC‐MSCs suppressed M1 polarization and induced M2 macrophage polarization (Figure 9B). HRMCs were incubated with high glucose (30 mM) medium. After 12, 24, 48, and 72 h of increased glucose, we changed the normal glucose (5.6 mM) medium and detected the mRNA expression of TGF‐β and collagen I (Figure 9C). We found more hours’ treatment of high glucose increased the expression of TGF‐β and collagen I expression. After 72 h’ stimulation of high glucose, the mRNA expression of TFG‐β increased compared with 48 h group. But there was no difference between 48 h group and 72 h group in collagen I expression (Figure 9C,D). To clarify the complex mechanisms by which UC‐MSC‐induced M2 macrophages improve the progression of DN, HRMCs were incubated in 30 mM for 48 h and cocultured with UC‐MSC‐induced M2 macrophages for another 48 h. We found UC‐MSC‐induced M2 macrophages reduced TGF‐β and collagen I expression (Figure 9E,G). Taken together, the results provided strong evidence that UC‐MSC‐induced M2 macrophages might be involved in the development of DN.

Figure 9UC‐MSC‐induced M2 macrophages ameliorated the ECM accumulation in HRMC: (A) human monocytic cell line THP‐1 cells were stained with CD80‐PE and CD206‐PE and then were analyzed by flow cytometry; (B) quantitative RT‐PCR analysis of gene expression in THP‐1 macrophages from three groups, results are presented relative to those of control group, set as 1; (C) quantitative RT‐PCR analysis of collagen I and TGF‐β expression in HRMC cultured with high glucose (30 mM). The results are presented relative to those of HRMC cultured with normal glucose (5.6 mM), set as 1; (D) photomicrographs of HRMC stained with anti‐collagen I (red) antibody from different groups according to the time of being exposed to high glucose (0, 12, 24, 48, and 72 h). Quantification of collagen I positive macrophages was determined by evaluating at least five random fields of each section. Results are presented relative to those of the 0 h group, set as 1; representative of three independent experiments; data are presented as mean ± SD from three independent experiments; quantitative data are presented in Supporting Information 2: Table S5. (E) Photomicrographs of HRMC stained with anti‐collagen I (red) antibody from control (cultured with normal glucose for 96 h), H (cultured with high glucose for 48 h and normal glucose for another 48 h), and M2 (cultured with high glucose for 48 h and normal glucose for another 48 h cocultured with UC‐MSC‐induced M2 macrophages groups). Quantification of collagen I positive macrophages was determined by evaluating at least five random fields of each section. Results are presented relative to those of the control group, set as 1; representative of three independent experiments; data are presented as mean ± SD from three independent experiments; quantitative data are presented in Supporting Information 2: Table S5. (F,G) Immunoblotting analysis of collagen I and TGF‐β in HRMC from the three groups. Protein levels are presented relative to β‐actin; representative of three independent experiments; data are presented as mean ± SD from three independent experiments; quantitative data are presented in Supporting Information 2: Table S5.

**Figure:**
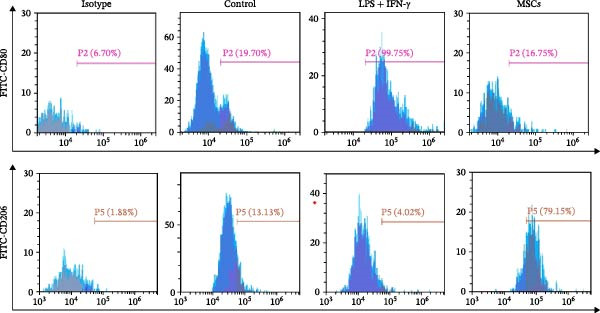
(A)

**Figure:**
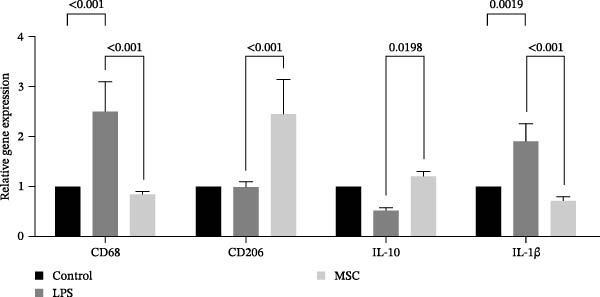
(B)

**Figure:**
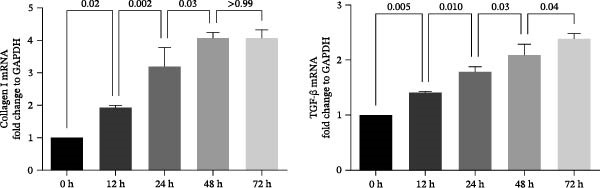
(C)

**Figure:**
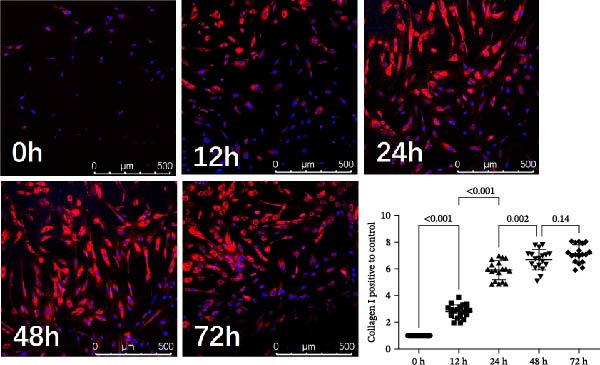
(D)

**Figure:**
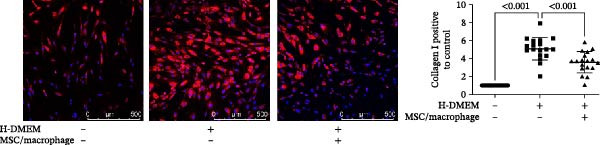
(E)

**Figure:**
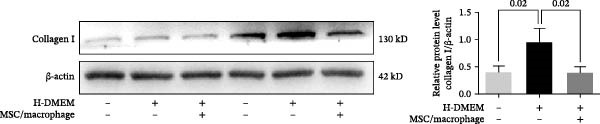
(F)

**Figure:**
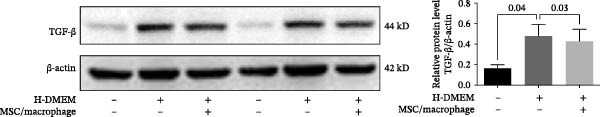
(G)

## 4. Discussion

Our current study provided a strong evidence that UC‐MSCs were involved directly in DN by inducing M2 macrophage polarization. MSCs have been lauded as a novel therapeutic strategy for diabetes mellitus and its associated complications because MSCs have several advantages, for instance, their ability to migrate to injured tissues, immune‐suppressive effects, and safely properties [31–33]. Previous studies demonstrated that dynamic tracking of UC‐MSCs following intravenous administration in mice model did not affect the blood biochemistry profiles of the liver, pancreas, kidney, and cardiac [24, 25]. In our study, we continuously observed the mice for 7–8 weeks after injecting human‐derived MSCs to check for clinical signs of immune rejection, such as weight loss, ruffled fur, reduced activity, or tissue inflammation at the injection site. No obvious abnormal symptoms were detected in the experimental mice during this period. Because relatively few MSCs migrated to the kidneys, previous studies concentrated on the immune‐regulatory function of MSCs, such as reducing oxidative stress, increasing the secretion of antiapoptotic cytokines, suppressing inflammation, producing anti‐inflammatory mediators, and producing growth factors, leading to the amelioration of ECM accumulation and renal damages [34, 35]. Despite the paracrine effects of MSCs on the prevention of DN having been previously documented, the molecular crosstalk mechanism between MSCs and macrophages remained unclear. Consistent with previous studies, MSC infusion ameliorated renal injury by suppressing inflammation, as demonstrated by the downregulation of IL‐1β, TNF‐α, and TGF‐β in our study. Notably, macrophages were the major inflammatory cell type in the development of DN. Similarly, the proportion of classically activated macrophage (M1) increased while the alternatively activated macrophage (M2) decreased in the kidney in DN. M1 macrophages are characterized by the overexpression of pro‐inflammatory cytokines; in contrast, M2 macrophages are considered to be involved in immune‐regulatory functions. Interestingly, we demonstrated that UC‐MSCs decreased the number of M1 macrophages but increased that of M2 macrophages in STZ‐induced diabetic rats. Furthermore, UC‐MSC‐induced M2 macrophages protect glomerular mesangial cells from high‐glucose toxicity in vitro. Therefore, the therapeutic effects of UC‐MSCs on DN were partially attributed to macrophage polarization phenotype.

Macrophages play a crucial role in inflammation, while distinct functional phenotypes of macrophages are acquired depending on the microenvironment. M1 macrophages are induced by toll‐like receptor (TLR) ligands and IFN‐γ and are characterized by the production of pro‐inflammatory factors, including TNF‐𝛼, IL‐1β, IL‐6, IL‐12, and proteolytic enzymes. M2 macrophages are induced by IL‐4 and IL‐13 and secrete anti‐inflammatory cytokines, such as TGF‐β, IL‐1 receptor antagonist, and IL‐10 [7–10]. Recent studies have demonstrated that M1 macrophages switch to M2 macrophages under certain circumstances [8, 36]. Interestingly, MSCs have been reported to induce M2 macrophage polarization according to the secretion of soluble factors, including PGE2, TSG‐6, IL‐6, IDO, and TGF‐β1 [17–19, 29]. Furthermore, IL‐4/IL‐13/IL‐4R𝛼 overexpression has been documented in M2 polarized macrophages [37]. The different cytokines secreted by MSCs may be related to diverse inflammatory environments. In our study, UC‐MSCs promoted the polarization of macrophages from M1 to M2 by secreting IL‐6, and blocking IL‐6 secretion inhibited the UC‐MSCs effect on M2 macrophage polarization. However, we found that IL‐4 and IL‐10 levels were extremely low in MSC‐mediated M2 macrophages, but the expression of IL‐4R𝛼 in macrophages increased significantly. Previous studies have indicated that IL‐4R𝛼 played a crucial role in M2 macrophage polarization [37]. Our results revealed that MSCs suppressed M1 macrophages and induced M2 macrophage polarization via IL‐6/IL‐4R𝛼.

Recent studies have revealed that different versions of M2 macrophages, including M2a, M2b, M2c, and M2d, had diverse functional states. In particular, M2a macrophages, induced by IL‐4 and IL‐13 (high Arg, CD163, and CD206 expression, low IL‐1, IL‐6, TNF‐𝛼, and TGF‐β expression), and M2b macrophages, induced by immune complexes and TLR or IL‐1R agonists (high IL‐1, IL‐6, and TNF‐𝛼 expression, low CD163, CD206, and TGF‐β expression), both exert immune‐regulatory functions and drive type II responses. M2c macrophages, induced by IL‐10 (high CD163, CD206, and TGF‐β expression, low IL‐1, IL‐6, and TNF‐𝛼 expression), are predominantly related to suppression of immune responses and tissue remodeling. M2d macrophages, induced by TLR ligands (high IL‐10 expression, low vascular endothelial growth factor (VEGF), IL‐12, and TNF‐𝛼 expression), play an important role in the development of tumor cell invasion and metastasis [38–40]. MSC‐mediated M2 macrophages were generally characterized by increased expression of CD206 and CD163 and reduced expression of IL‐1β, TNF‐𝛼, and TGF‐β, suggesting that MSCs may induce M2a phenotype macrophage polarization.

Mesangial expansion is a characteristic feature of DN and closely correlates with ECM deposition and inflammation, leading to renal decline. Mesangial cells exposed to high glucose concentrations increase the expression of collagen and fibronectin and the secretion of cytokines, such as TGF‐β, connective tissue growth factor (CTGF), VEGF, and MCP‐1 [41]. In our study, MSC‐mediated M2 macrophages inhibited collagen I/IV, TGF‐β, and IL‐1β in mesangial cells in high glucose conditions. Interestingly, MCP‐1 was also decreased by M2 macrophages in vivo and in vitro to reduce the infiltration of monocytes and macrophages. In addition, Nox4 is a molecule that plays a key role in TGF‐β1‐driven fibrosis, and it is the main isoform expressed in the mesangial cells and participates in mesangial matrix expansion in DN. Nox‐4 contributes to high glucose‐induced mitochondrial ROS production in mesangial cells and induces ECM accumulation via TGF‐β signaling, which is a key regulator of ECM deposition that acts by enhancing the collagen and fibronectin, as well as by inhibiting ECM degradation [42, 43]. In our study, Nox4 expression increased in LPS‐stimulated M1 macrophages group but decreased in UC‐MSC‐induced M2 macrophages group. And UC‐MSC‐induced M2 macrophages downregulated TGF‐β and collagen I expression. So MSC‐mediated M2 macrophages may inhibit Nox‐4/TGF‐β/collagen I signaling in mesangial cells.

## 5. Conclusions

In conclusion, the UC‐MSC infusion reduced the infiltration of M1 macrophages and increased the infiltration of M2 macrophages in the glomerulus, thereby attenuating histopathological renal damage and improving renal inflammation and fibrosis in DN. These results provide a theoretical basis for the use of MSCs in clinical treatment of DN in patients with diabetes mellitus in the future.

**Nomenclature**
M1: Classically activated macrophages
M2: Alternatively activated macrophages
MSCs: Mesenchymal stem cells
DN: Diabetic nephropathy
UC‐MSCs: Human umbilical cord mesenchymal stem cells
LPS: Lipopolysaccharides
IL‐4Rα: Interleukin‐4 receptor alpha chain
IL‐6: Interleukin‐6
HBZY‐1: Rat glomerular mesangial cells
HRMC: Human renal mesangial cells
TNF‐α: Tumor necrosis factor α
TGF‐β: Transforming growth factor β
MCP‐1: Monocyte chemoattractant protein‐1
GBM: Glomerular basement membrane
STZ: Streptozotocin
HFD: High‐fat diet
NCD: Normal‐chow diet
T2DM: Type 2 diabetes mellitus
IPGTTs: Intraperitoneal glucose tolerance tests
IPITTs: Insulin tolerance tests
PBS: Phosphate‐buffered saline
qRT‐PCR: Quantitative real‐time reverse transcriptase polymerase chain reaction
ELISA: Enzyme‐linked immunosorbent assay
NA: Neutralizing antibody
IL‐1β: Interleukin‐1β
THP‐1: Tohoku Hospital Pediatrics‐1 cells
ACR: Albumin creatine ratio
H&E: Hematoxylin and eosin
PAS: Periodic acid Schiff
TSG‐6: TNF‐α‐stimulated gene 6
IDO: Indoleamine 2,3‐dioxygenase
TEM: Transmission electron microscopy
ECM: Extracellular matrix
α‐SMA: α‐Smooth muscle actin
EP‐4: Prostaglandin E receptor 4
STAT3: Signal transducer and activator of transcription 3
iNOS: Inducible nitric oxide synthase
Arg 1: Arginase 1
PGE2: Prostaglandin E2
NOX‐4: NADPH oxidase 4
TLR: Toll‐like receptor
IFN‐γ: Interferon γ
CTGF: Connective tissue growth factor
VEGF: Vascular endothelial growth factor
FACS: Fluorescence‐activated cell sorting.

## Author Contributions

Linxi Zhang, Songyan Yu, and Yu Cheng contributed to the conception and design, provision of study material, collection of data, data analysis and interpretation, and manuscript writing. Xiafang Lin, Zhengyuan Gong, Jing Xue, and Bing Li contributed to the provision of study material and collection of data. Yaqi Yin and Junyan Zou contributed to the collection of data and data analysis and interpretation. Rui Wei contributed to the provision of study material and data analysis and interpretation. Yiming Mu and Tianpei Hong contributed to the conception and design, financial support, manuscript writing, and final approval of manuscript.

## Funding

This work was supported by the National Natural Science Foundation of China (Grants 81870578, 81900754, 82170875, and 82100861).

## Disclosure

An earlier version of this paper is published in a preprint [44]. Linxi Zhang, Songyan Yu, Yu Cheng, Zhengyuan Gong, Jing Xue, Bing Li, Yaqi Yin, Junyan Zou, Rui Wei, Tianpei Hong, Yiming Mu, Mesenchymal stem cells polarize macrophages to an anti‐inflammatory phenotype to ameliorate diabetic nephropathy, 2022]. According to the following link: https://doi.org/10.21203/rs.3.rs-1965742/v1. All authors read and approved the final manuscript.

## Ethics Statement

All animal experiment protocols were approved by the Medical Ethics Committee of the Chinese PLA General Hospital.

## Consent

All subjects provided informed consent. Ethics committee of the First Medical Center of Chinese PLA General Hospital approved the study.

## Conflicts of Interest

The authors declare no conflicts of interest.

## Supporting Information

Additional supporting information can be found online in the Supporting Information section.

## Supporting information


**Supporting Information 1** Figure S1. (A) Body weight were detected at the age of 8, 10, 12, 14 and 16 weeks. NCD: Normal chow diet, HFD: High fat diet. Maintaining the body weight of HFD to 600 g, a low dose of STZ (22 mg/kg) was injected to obtain T2D model. (B) Blood glucose were measured after STZ administration from N and DM group. (C) Blood glucose after IPGTT test in these two groups. IPGTT: Intraperitoneal glucose tolerance test. (D) Blood glucose after IPITT test in these two groups. IPITT: Intraperitoneal insulin tolerance test. N: Normal, DM: Type 2 diabetes. Data were presented as mean ± SD.  ^∗^ *p* < 0.05;  ^∗∗^ *p* < 0.01;  ^∗∗∗^ *p* < 0.001.

**Supporting Information 2** Table S1. Demographics of umbilical cord donors. Table S2. Information of antibodies for immunohistochemistry and immunofluorescence staining. Table S3. Information of antibodies for immunoblotting tests. Table S4. Primer sequences for qRT‐PCR. Table S5. Quantitative data of all replicates for in vitro experiments.

**Supporting Information 3** Figure S2. (A) UC‐MSCs were cultured with LPS‐stimulated macrophages (M1) for 24 h, and the gene expression of the factors secreted by UC‐MSCs was detected by quantitative RT‐PCR analysis. The control group was UC‐MSCs cultured alone; results are presented relative to those of the control group, set as 1. Results were presented as the means ± SD. (B) Enzyme‐linked immunosorbent assays of IL‐4 and IL‐13 in the medium of UC‐MSCs cocultured with LPS‐stimulated macrophages (M1). The control group was UC‐MSCs cultured alone; Results were presented as the means ± SD.

## Data Availability

The data used to support the findings of this study are included within the article.
