# Supplementary figures and images for: Mesenchymal Stem Cells Polarize Macrophages to an Anti‐Inflammatory Phenotype to Ameliorate Diabetic Nephropathy
Source: Stem Cells Int. 2026 Feb 18;2026:6684410. doi: 10.1155/sci/6684410 (PMC12916875; doi:10.1155/sci/6684410)

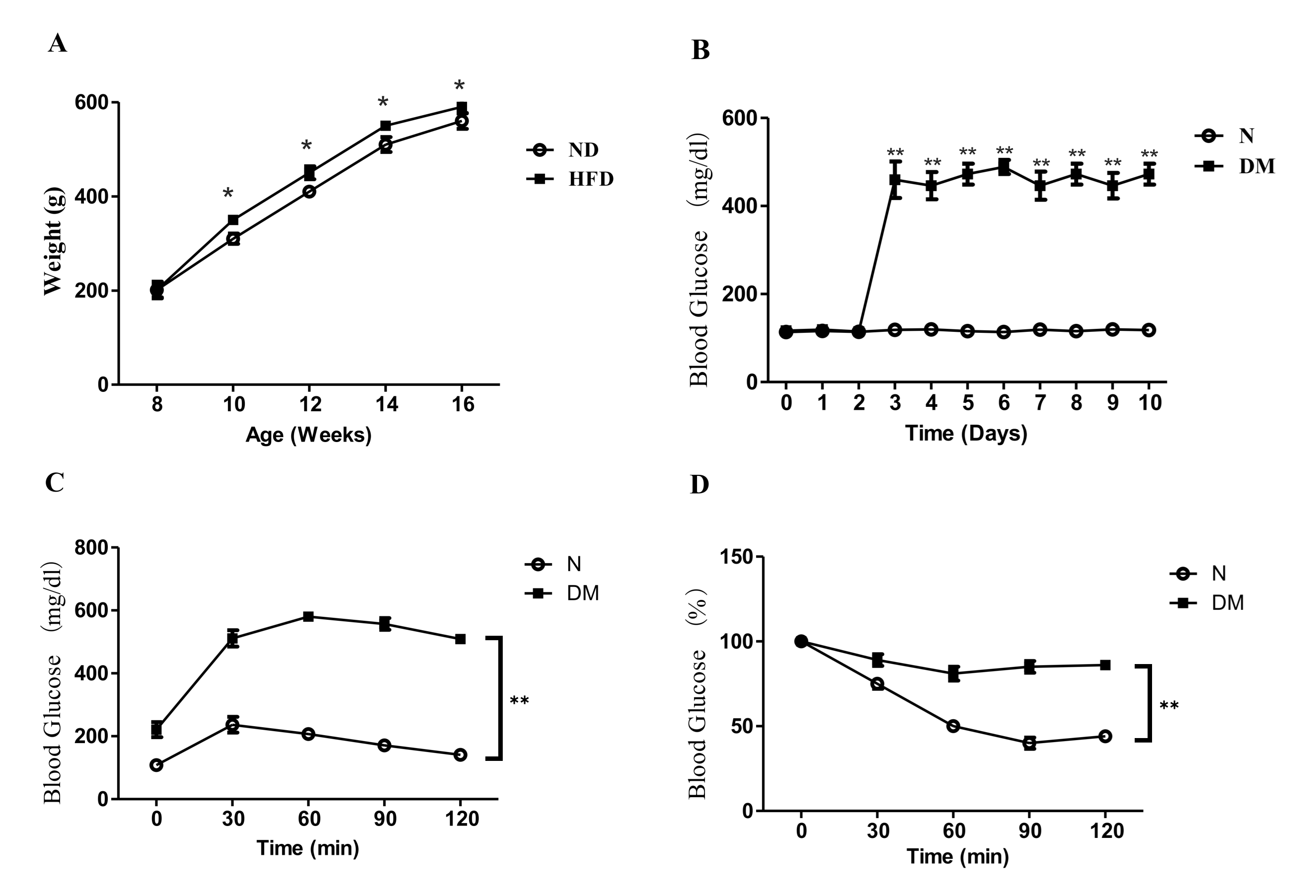

Supplement: Supplementary file 1 — Supporting Information 1 Figure S1. (A) Body weight were detected at the age of 8, 10, 12, 14 and 16 weeks. NCD: Normal chow diet, HFD: High fat diet. Maintaining the body weight of HFD to 600 g, a low dose of STZ (22 mg/kg) was injected to obtain T2D model. (B) Blood glucose were measured after STZ administration from N and DM group. (C) Blood glucose after IPGTT test in these two groups. IPGTT: Intraperitoneal glucose tolerance test. (D) Blood glucose after IPITT test in these two groups. IPITT: Intraperitoneal insulin tolerance test. N: Normal, DM: Type 2 diabetes. Data were presented as mean ± SD. ∗ p < 0.05; ∗∗ p < 0.01; ∗∗∗ p < 0.001. [file SCI-2026-6684410-s001.tif]

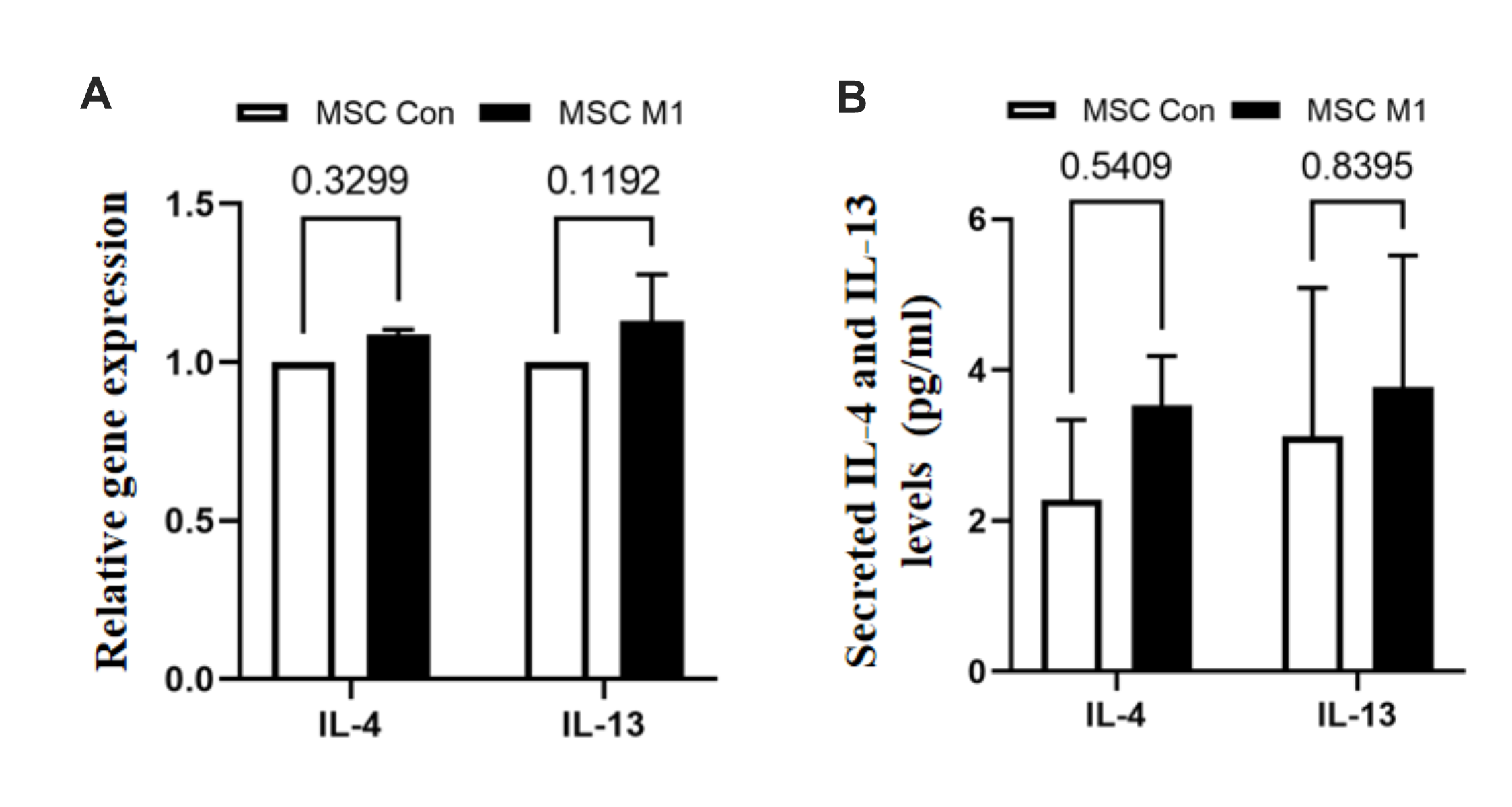

Supplement: Supplementary file 3 — Supporting Information 3 Figure S2. (A) UC‐MSCs were cultured with LPS‐stimulated macrophages (M1) for 24 h, and the gene expression of the factors secreted by UC‐MSCs was detected by quantitative RT‐PCR analysis. The control group was UC‐MSCs cultured alone; results are presented relative to those of the control group, set as 1. Results were presented as the means ± SD. (B) Enzyme‐linked immunosorbent assays of IL‐4 and IL‐13 in the medium of UC‐MSCs cocultured with LPS‐stimulated macrophages (M1). The control group was UC‐MSCs cultured alone; Results were presented as the means ± SD. [file SCI-2026-6684410-s002.tif]
