# Supplementary material for: Mesenchymal Stem Cells Polarize Macrophages to an Anti‐Inflammatory Phenotype to Ameliorate Diabetic Nephropathy
Source: Stem Cells Int. 2026 Feb 18;2026:6684410. doi: 10.1155/sci/6684410 (PMC12916875; doi:10.1155/sci/6684410)
Supplement: Supplementary file 2 — Supporting Information 2 Table S1. Demographics of umbilical cord donors. Table S2. Information of antibodies for immunohistochemistry and immunofluorescence staining. Table S3. Information of antibodies for immunoblotting tests. Table S4. Primer sequences for qRT‐PCR. Table S5. Quantitative data of all replicates for in vitro experiments. [file SCI-2026-6684410-s003.docx]

**Table S1 Demographics of umbilical cord donors.**

|  | 1 | 2 | 3 | 4 | 5 | 6 | 7 | 8 |
| --- | --- | --- | --- | --- | --- | --- | --- | --- |
| Ages（years） | 28 | 27 | 34 | 33 | 25 | 29 | 33 | 30 |
| BMI（kg/m^2^） | 24.35 | 22.78 | 23.45 | 21.34 | 21.78 | 21.90 | 22.69 | 23.29 |
| Gestational weeks | 38 | 39 | 40 | 37 | 39 | 40 | 41 | 39 |
| Parity | 1 | 1 | 1 | 2 | 1 | 2 | 1 | 1 |
| Spontaneous labor | Yes | Yes | No | Yes | Yes | No | Yes | Yes |
| Cesarean delivery | No | No | Yes | No | No | Yes | No | No |
| Fasting blood glucose  (mmol/L) | - | 4.3 | - | 4.9 | 4.4 | 4.1 | - | 4.9 |
| Random blood glucose  (mmol/L) | 6.3 | - | 6.5 | - | - | - | 5.9 | - |

**Table S2 Information of antibodies for immunohistochemistry and immunofluorescence staining.**

|  | Company | Produce code | Dilution | Host species |
| --- | --- | --- | --- | --- |
| Anti-Collagen I antibody | Abcam | Ab34710 | 1:200 | Rabbit |
| Anti-Collagen IV antibody | Abcam | Ab6586 | 1:200 | Rabbit |
| Anti-Fibronectin antibody | Abcam | Ab2413 | 1:200 | Rabbit |
| Anti- IL-1 beta antibody | Abcam | Ab9722 | 1:200 | Rabbit |
| Anti-TNF alpha antibody | Abcam | Ab6671 | 1:100 | Rabbit |
| Anti-TGF beta 1 antibody | Abcam | Ab92486 | 1:200 | Rabbit |
| Anti-CD68 antibody | Abcam | Ab955 | 1:200 | Mouse |
| Anti-Mannose Receptor（CD206） | Abcam | Ab64693 | 1:100 | Rabbit |
| Anti-CD11c antibody | Abcam | Ab11029 | 1:100 | Mouse |
| Mouse anti Rat CD163 | Bio-Rad | MCA342R | 1:100 | Mouse |
| F4/80 (A-19) | Santa Cruz | Sc-26642 | 1:100 | Mouse |
| Anti-liver Arginase antibody | Abcam | Ab91279 | 1:100 | Rabbit |
| Anti-iNOS antibody | Abcam | Ab49999 | 1:100 | Mouse |
| Anti-Collagen I antibody | Abcam | Ab138492 | 1:200 | Rabbit |
| PE/Cyanine7 anti-human CD68 Antibody | Biolegend | 333816 | - | Mouse |
| FITC anti-human CD80 Antibody | Biolegend | 305206 | - | Mouse |
| PE anti-human CD206 (MMR) Antibody | Biolegend | 321106 | - | Mouse |

**Table S3 Information of antibodies for immunoblotting tests.**

|  | Company | Produce code | Dilution | Host species |
| --- | --- | --- | --- | --- |
| β-actin antibody | ZSGB-BIO | TA-09 | 1:2000 | Mouse |
| Anti-Collagen I antibody | Abcam | Ab260043 | 1:1000 | Rabbit |
| Anti-TNF alpha antibody | Abcam | Ab6671 | 1:1000 | Rabbit |
| Anti-TGF beta 1 antibody | Abcam | Ab215715 | 1:1000 | Rabbit |
| Anti-liver Arginase antibody | Abcam | Ab91279 | 1:1000 | Rabbit |
| IL-4Rα（S-20） | Santa Cruz | Sc-686 | 1:200 | Rabbit |
| Anti-NADPH oxidase 4 antibody | Immunoway | YM8112 | 1:1000 | Rabbit |

**Table S4 Primer sequences for qRT-PCR.**

| Genes (Rat) | Primer sequences（5’— 3’） |
| --- | --- |
| Collagen I | For：GATGGACTCAACGGTCTCCC |
|  | Rev：CGGCCACCATCTTGAGACTT |
| Collagen IV | For：CTTCGTTGGCCTCTGTTTGC |
|  | Rev：TTCTTGTGGAGTTCTCGCCC |
| Acta2 | For：CATCCGACCTTGCTAACGGA |
|  | Rev：TGGCATGAGGCAGAGCATAG |
| β-actin | For：ACGGTCAGGTCATCACTATCG |
|  | Rev：GGCATAGAGGTCTTTACGGATG |
| TNF-α | For：ATGGGCTCCCTCTCATCAGTTC |
|  | Rev：CTCCTCCGCTTGGTGGTTTG |
| TGF-β | For：ATACGCCTGAGTGGCTGTCT |
|  | Rev：TTGGGACTGATCCCATTGAT |
| CD68 | For：TGTACCTGACCCAGGGTGGAA |
|  | Rev：GAATCCAAAGGTAAGCTGTCCGTAA |
|  | Rev：GAAGATGTCAAACTCATTCATGGCC |
| CCL2 | For：TTAATGCCCCACTCACCTGC |
|  | Rev：CCTTATTGGGGTCAGCACAGA |
| EP4 | For：GCACCTCTCAGACCCTCCTA |
|  | Rev：AAGTTCTCAGCGAGGTGGTG |
| STAT3 | For：AAGCTGACCCAGGTAGTGCT |
|  | Rev：TCCATGTCAAACGTGAGCGA |
| CD206 | For：ACTGCGTGGTGATGAAAGG |
|  | Rev：TAACCCAGTGGTTGCTCACA |
| iNOS | For：CCAACCTGCAGGTCTTCGATG |
|  | Rev：GTCGATGCACAACTGGGTGAAC |
| CD163 | For：TGTAGTTCATCATCTTCGGTCC |
|  | Rev：CACCTACCAAGCGGAGTTGAC |
| IL-6 | For：CCGGAGAGGAGAATTCACAG |
|  | Rev：TGACAGTGCATCGCTGTTC |
| IL-1β | For：TACCTATGTCTTGCCCGTGGAG |
|  | Rev：ATCATCCCACGAGTCACAGAGG |
| Cox-2 | For：GTATCGGGTTTTGAACGGCG |
|  | Rev：GGACCAAGCCAAAATGCTCG |
| IL-10 | For：ATGGCCCAGAAATCAAGGAGC |
|  | Rev：GAAGATGTCAAACTCATTCATGGCC |

| Genes (Human) | Primer sequence（5’— 3’） |
| --- | --- |
| IL-6 | For：GGTACATCCTCGACGGCATCT |
|  | Rev：GTGCCTCTTTGCTGCTTTCAC |
| β-actin | For：GAGGTGAAGAGCATCGGGG |
|  | Rev：TCCTGGGTCTCTCCTGGTTT |
| IDO | For：GCCAGCTTCGAGAAAGAGTTG |
|  | Rev：ATCCCAGAACTAGACGTGCAA |
| TGF-β1 | For：TGGTGGAAACCCACAACGAAGA |
|  | Rev：GCAACACGGGTTCAGGTA |
| TSG6 | For：GCTAGAGGCAGCCAGAAAAA |
|  | Rev：ATCCAACTCTGCCCTTAGCC |
| CD86 | For：CTGCTCATCTATACACGGTTACC |
|  | Rev：GGAAACGTCGTACAGTTCTGTG |
| CD206 | For：TTCGGACACCCATCGGAATTT |
|  | Rev：CACAAGCGCTGCGTGGAT |
| IL-10 | For：AAGACCCAGACATCAAGGCG |
|  | Rev：AGGCATTCTTCACCTGCTCC |
| IL-1β | For：CAGTGGCAATGAGGATGACTTGTTCT |
|  | Rev：CTGTAGTGGTGGTCGGAGATTCGTA |
| Collagen-I | For：GCGAGAGCATGACCGATGGATTC |
|  | Rev：GCCTTCTTGAGGTTGCCAGTCTG |

**Table S5 Quantitative data of all replicates for in vitro experiments.**

1. Figure 6C Immunofluorescence of Arg 1 and iNOS in the control, LPS and MSC groups. Quantification of Arg 1 positive or iNOS positive macrophages was determined by evaluating six random fields of each section.

| Number of Arg 1  positive cell(%) | Con | LPS | MSC |
| --- | --- | --- | --- |
| replicate 1-1 | 40.382 | 37.873 | 74.287 |
| replicate 1-2 | 52.621 | 39.763 | 70.973 |
| replicate 1-3 | 50.653 | 48.365 | 80.386 |
| replicate 1-4 | 36.652 | 32.375 | 72.372 |
| replicate 1-5 | 41.072 | 39.090 | 82.375 |
| replicate 1-6 | 42.386 | 38.982 | 74.376 |
| replicate 2-1 | 37.763 | 35.271 | 77.376 |
| replicate 2-2 | 37.762 | 35.286 | 75.725 |
| replicate 2-3 | 32.038 | 32.976 | 79.376 |
| replicate 2-4 | 31.083 | 29.036 | 84.820 |
| replicate 2-5 | 54.972 | 48.651 | 69.972 |
| replicate 2-6 | 36.862 | 33.761 | 80.387 |
| replicate 3-1 | 33.862 | 33.324 | 85.273 |
| replicate 3-2 | 50.381 | 41.273 | 79.876 |
| replicate 3-2 | 51.376 | 39.361 | 70.365 |
| replicate 3-4 | 34.762 | 33.761 | 74.862 |
| replicate 3-5 | 47.762 | 43.812 | 80.281 |
| replicate 3-6 | 46.261 | 37.931 | 72.084 |

| Number of iNOS  positive cell(%) | Con | LPS | MSC |
| --- | --- | --- | --- |
| replicate 1-1 | 20.028 | 42.972 | 20.927 |
| replicate 1-2 | 24.263 | 45.283 | 17.082 |
| replicate 1-3 | 18.037 | 40.820 | 20.236 |
| replicate 1-4 | 20.371 | 45.083 | 16.373 |
| replicate 1-5 | 17.381 | 40.383 | 17.950 |
| replicate 1-6 | 24.92 | 44.846 | 23.371 |
| replicate 2-1 | 22.023 | 43.273 | 19.376 |
| replicate 2-2 | 19.721 | 45.386 | 18.037 |
| replicate 2-3 | 22.234 | 49.72 | 19.361 |
| replicate 2-4 | 24.293 | 43.971 | 23.271 |
| replicate 2-5 | 17.092 | 42.974 | 22.381 |
| replicate 2-6 | 23.932 | 40.286 | 23.961 |
| replicate 3-1 | 25.292 | 38.972 | 16.381 |
| replicate 3-2 | 26.301 | 30.971 | 19.470 |
| replicate 3-2 | 24.381 | 40.183 | 20.710 |
| replicate 3-4 | 26.492 | 38.436 | 18.302 |
| replicate 3-5 | 20.983 | 44.842 | 23.813 |
| replicate 3-6 | 21.103 | 47.381 | 16.371 |

1. Figure 6D Immunoblotting analysis of Arg 1 in the three groups. Relative protein level is quantified by ratio of Arg1 to β-actin.

| Relative protein level  Arg1 to β-actin | replicate  1 | replicate  2 | replicate  3 |
| --- | --- | --- | --- |
| Con | 0.206813 | 0.137503 | 0.303820 |
| LPS | 0.418516 | 0.371092 | 0.323739 |
| MSC | 1.203997 | 1.309348 | 1.327364 |

1. Figure 7E Immunofluorescence of Arg 1 positive macrophages. Quantification of Arg 1 positive macrophages was determined by evaluating five random fields of each section.

| Number of Arg 1  positive cell(%) | Con | LPS | LPS+MSC | LPS+MSC  +IgG | LPS+MSC  +IL-6NA |
| --- | --- | --- | --- | --- | --- |
| replicate 1-1 | 40.721 | 37.971 | 80.082 | 78.752 | 38.871 |
| replicate 1-2 | 45.927 | 43.983 | 84.872 | 75.653 | 36.872 |
| replicate 1-3 | 33.092 | 32.661 | 78.862 | 80.002 | 40.038 |
| replicate 1-4 | 47.711 | 36.642 | 75.092 | 82.652 | 36.652 |
| replicate 1-5 | 44.624 | 41.003 | 82.002 | 79.862 | 32.653 |
| replicate 2-1 | 42.989 | 35.082 | 74.862 | 76.902 | 38.861 |
| replicate 2-2 | 34.081 | 35.083 | 80.972 | 78.762 | 30.861 |
| replicate 2-3 | 45.160 | 43.971 | 79.362 | 78.032 | 34.762 |
| replicate 2-4 | 37.813 | 35.872 | 83.072 | 77.776 | 35.876 |
| replicate 2-5 | 41.182 | 41.037 | 78.072 | 81.020 | 31.923 |
| replicate 3-1 | 53.081 | 48.871 | 76.372 | 75.922 | 39.081 |
| replicate 3-2 | 35.871 | 35.462 | 84.027 | 80.372 | 41.027 |
| replicate 3-2 | 38.087 | 34.998 | 86.092 | 75.282 | 37.862 |
| replicate 3-4 | 39.082 | 38.982 | 77.962 | 81.028 | 33.232 |
| replicate 3-5 | 45.661 | 42.972 | 72.083 | 79.201 | 34.361 |

1. Figure 7F Immunoblotting analysis of IL-4Rα in the three groups. Relative protein level is quantified by ratio of IL-4Rα to β-actin.

| Relative protein level  IL-4Rα to β-actin | replicate  1 | replicate  2 | replicate  3 |
| --- | --- | --- | --- |
| LPS | 0.618842 | 0.654893 | 0.452938 |
| LPS+MSC+IgG | 1.773391 | 1.883764 | 1.303847 |
| LPS+MSC+IL-6 NA | 0.803745 | 0.907542 | 0.603729 |

1. Figure 8A Quantification of collagen I positive was determined by evaluating five random fields of each section. Results are presented relative to those of the control group, set as 1.

| Collagen I positive to  12h L-DMED | 12h | | 24h | |
| --- | --- | --- | --- | --- |
|  | L-DMEM | H-DMEM | L-DMEM | H-DMEM |
| replicate 1-1 | 1 | 4.092 | 2.209 | 10.732 |
| replicate 1-2 | 1 | 4.121 | 2.698 | 10.300 |
| replicate 1-3 | 1 | 4.227 | 2.521 | 10.939 |
| replicate 1-4 | 1 | 3.994 | 2.421 | 8.092 |
| replicate 1-5 | 1 | 2.624 | 1.392 | 10.982 |
| replicate 2-1 | 1 | 4.349 | 2.521 | 10.730 |
| replicate 2-2 | 1 | 4.082 | 1.981 | 9.872 |
| replicate 2-3 | 1 | 3.980 | 1.491 | 9.998 |
| replicate 2-4 | 1 | 4.320 | 2.239 | 11.021 |
| replicate 2-5 | 1 | 4.184 | 1.890 | 10.812 |
| replicate 3-1 | 1 | 3.650 | 2.002 | 8.982 |
| replicate 3-2 | 1 | 2.745 | 2.106 | 8.340 |
| replicate 3-2 | 1 | 5.294 | 1.983 | 12.291 |
| replicate 3-4 | 1 | 3.876 | 1.642 | 11.932 |
| replicate 3-5 | 1 | 4.022 | 1.713 | 9.812 |

1. Figure 8C Quantification of collagen I positive macrophages was determined by evaluating six random fields of each section. Results are presented relative to those of the control group, set as 1.

| Collagen I positive  to control | Con | LPS | MSC |
| --- | --- | --- | --- |
| replicate 1-1 | 1 | 4.132 | 1.909 |
| replicate 1-2 | 1 | 4.509 | 2.109 |
| replicate 1-3 | 1 | 3.712 | 2.129 |
| replicate 1-4 | 1 | 4.207 | 1.883 |
| replicate 1-5 | 1 | 3.915 | 2.228 |
| replicate 1-6 | 1 | 4.628 | 2.091 |
| replicate 2-1 | 1 | 4.121 | 1.972 |
| replicate 2-2 | 1 | 3.771 | 2.091 |
| replicate 2-3 | 1 | 4.391 | 2.911 |
| replicate 2-4 | 1 | 3.981 | 2.931 |
| replicate 2-5 | 1 | 4.201 | 1.928 |
| replicate 2-6 | 1 | 3.898 | 1.890 |
| replicate 3-1 | 1 | 4.092 | 2.381 |
| replicate 3-2 | 1 | 3.764 | 2.472 |
| replicate 3-2 | 1 | 3.519 | 2.648 |
| replicate 3-4 | 1 | 4.729 | 2.091 |
| replicate 3-5 | 1 | 3.670 | 2.742 |
| replicate 3-6 | 1 | 3.908 | 2.391 |

1. Figure 8D Immunoblotting analysis of NOX-4, TGF-βand collagen I in HBZY-1 from the three groups. Protein levels are presented relative to β-actin.

| Relative protein level  to β-actin | Con | H-DMEM | H-DMEM  MSC/macrophage |
| --- | --- | --- | --- |
| Nox-4/β-actin replicate 1 | 0.461489 | 0.852195 | 0.490122 |
| Nox-4/β-actin replicate 2 | 0.406828 | 0.937810 | 0.538194 |
| Nox-4/β-actin replicate 3 | 0.423201 | 0.902632 | 0.501721 |
| Nox-4/β-actin replicate 4 | 0.675418 | 0.916789 | 0.855220 |
| TGF-β/β-actin replicate 1 | 0.217919 | 0.859865 | 0.352004 |
| TGF-β/β-actin replicate 2 | 0.302718 | 0.901426 | 0.402416 |
| TGF-β/β-actin replicate 3 | 0.271564 | 0.778362 | 0.299817 |
| TGF-β/β-actin replicate 4 | 0.466211 | 0.497999 | 0.430591 |
| collagen 1/β-actin replicate 1 | 0.587452 | 0.700281 | 0.577344 |
| collagen 1/β-actin replicate 2 | 0.451691 | 1.031930 | 0.452781 |
| collagen 1/β-actin replicate 3 | 0.302183 | 0.897251 | 0.399835 |
| collagen 1/β-actin replicate 4 | 0.480271 | 1.197103 | 0.388719 |

1. Figure 9D Quantification of collagen I positive macrophages was determined by evaluating six random fields of each section. Results are presented relative to those of the 0h group, set as 1.

| Collagen I positive  to control | 0h | 12h | 24h | 48h | 72h |
| --- | --- | --- | --- | --- | --- |
| replicate 1-1 | 1 | 3.423 | 5.834 | 7.834 | 8.054 |
| replicate 1-2 | 1 | 3.082 | 6.323 | 6.234 | 7.236 |
| replicate 1-3 | 1 | 2.737 | 6.975 | 7.123 | 7.265 |
| replicate 1-4 | 1 | 2.223 | 5.973 | 6.546 | 7.936 |
| replicate 1-5 | 1 | 2.545 | 5.934 | 6.334 | 6.586 |
| replicate 1-6 | 1 | 2.928 | 6.822 | 7.642 | 6.946 |
| replicate 2-1 | 1 | 2.926 | 6.835 | 7.126 | 8.035 |
| replicate 2-2 | 1 | 1.974 | 4.862 | 6.972 | 7.083 |
| replicate 2-3 | 1 | 3.862 | 6.482 | 7.772 | 8.073 |
| replicate 2-4 | 1 | 2.279 | 5.972 | 6.772 | 7.372 |
| replicate 2-5 | 1 | 2.974 | 5.028 | 5.987 | 6.362 |
| replicate 2-6 | 1 | 1.978 | 5.823 | 6.743 | 7.073 |
| replicate 3-1 | 1 | 2.533 | 4.875 | 5.083 | 5.903 |
| replicate 3-2 | 1 | 3.072 | 6.734 | 7.074 | 7.083 |
| replicate 3-2 | 1 | 2.553 | 5.973 | 6.373 | 6.982 |
| replicate 3-4 | 1 | 2.840 | 5.752 | 6.072 | 6.525 |
| replicate 3-5 | 1 | 3.281 | 4.872 | 5.432 | 6.083 |
| replicate 3-6 | 1 | 2.876 | 5.082 | 7.063 | 7.973 |

1. Figure 9E Quantification of collagen I positive macrophages was determined by evaluating six random fields of each section. Results are presented relative to those of the control group, set as 1.

| Collagen I positive  to control | Con | H-DMEM | H-DMEM  MSC/macrophage |
| --- | --- | --- | --- |
| replicate 1-1 | 1 | 5.032 | 3.702 |
| replicate 1-2 | 1 | 4.921 | 3.729 |
| replicate 1-3 | 1 | 5.512 | 3.529 |
| replicate 1-4 | 1 | 6.271 | 4.359 |
| replicate 1-5 | 1 | 5.390 | 3.349 |
| replicate 1-6 | 1 | 5.610 | 3.705 |
| replicate 2-1 | 1 | 3.923 | 2.014 |
| replicate 2-2 | 1 | 4.293 | 3.828 |
| replicate 2-3 | 1 | 3.472 | 1.937 |
| replicate 2-4 | 1 | 5.028 | 3.863 |
| replicate 2-5 | 1 | 4.399 | 2.972 |
| replicate 2-6 | 1 | 2.038 | 1.083 |
| replicate 3-1 | 1 | 7.937 | 5.863 |
| replicate 3-2 | 1 | 6.037 | 4.766 |
| replicate 3-2 | 1 | 5.047 | 4.872 |
| replicate 3-4 | 1 | 4.950 | 3.017 |
| replicate 3-5 | 1 | 5.973 | 2.942 |
| replicate 3-6 | 1 | 6.234 | 5.082 |

1. Figure 9F-G Immunoblotting analysis of collagen I and TGF-β in human mesangial cells from the three groups. Protein levels are presented relative to β-actin.

| Relative protein level  to β-actin | Con | H-DMEM | H-DMEM  MSC/macrophage |
| --- | --- | --- | --- |
| TGF-β/β-actin replicate 1 | 0.201367 | 0.560837 | 0.505931 |
| TGF-β/β-actin replicate 2 | 0.158235 | 0.529666 | 0.487913 |
| TGF-β/β-actin replicate 3 | 0.232497 | 0.335052 | 0.289961 |
| collagen 1/β-actin replicate 1 | 0.536528 | 1.188077 | 0.436661 |
| collagen 1/β-actin replicate 2 | 0.334655 | 0.983094 | 0.469893 |
| collagen 1/β-actin replicate 3 | 0.334665 | 0.685649 | 0.262614 |

**Supplementary Figure S1**

1. Body weight were detected at the age of 8, 10, 12, 14 and 16 weeks. NCD: Normal chow diet, HFD: High fat diet. Maintaining the body weight of HFD to 600g, a low dose of STZ (22mg/kg) was injected to obtain T2D model.
2. Blood glucose were measured after STZ administration from N and DM group.
3. Blood glucose after IPGTT test in these two groups. IPGTT: Intraperitoneal glucose tolerance test.
4. Blood glucose after IPITT test in these two groups. IPITT: Intraperitoneal insulin tolerance test.

N: Normal, DM: Type 2 diabetes. Data were presented as mean ± SD. *p<0.05; **p<0.01; ***p<0.001.

**Supplementary Figure S2**

1. UC-MSCs were cultured with LPS-stimulated macrophages (M1) for 24 h, and

the gene expression of the factors secreted by UC-MSCs was detected by quantitative RT-PCR analysis. The control group was UC-MSCs cultured alone; results are presented relative to those of the control group, set as 1. Results were presented as the means ± SD.

1. Enzyme-linked immunosorbent assays of IL-4 and IL-13 in the medium of

UC-MSCs co-cultured with LPS-stimulated macrophages (M1) . The control group was UC-MSCs cultured alone; Results were presented as the means ± SD.
